# TRIP6 functions in brain ciliogenesis

**DOI:** 10.1038/s41467-021-26057-6

**Published:** 2021-10-07

**Authors:** Shalmali Shukla, Ronny Haenold, Pavel Urbánek, Lucien Frappart, Shamci Monajembashi, Paulius Grigaravicius, Sigrun Nagel, Woo Kee Min, Alicia Tapias, Olivier Kassel, Heike Heuer, Zhao-Qi Wang, Aspasia Ploubidou, Peter Herrlich

**Affiliations:** 1 Leibniz Institute on Aging – Fritz Lipmann Institute (FLI), Beutenbergstr. 11, 07745 Jena, Germany; 2 INSERM, Oncogenèse et Progression Tumorale, Université Claude Bernard Lyon I, 69373 Lyon, France; 3 Karlsruhe Institute of Technology (KIT), Institute for Biological and Chemical Systems-Biological Information Processing (IBCS-BIP), 76131 Karlsruhe, Germany; 4 Department of Endocrinology, University of Duisburg-Essen, 45147 Essen, Germany

**Keywords:** Cell biology, Diseases

## Abstract

TRIP6, a member of the ZYXIN-family of LIM domain proteins, is a focal adhesion component. *Trip6* deletion in the mouse, reported here, reveals a function in the brain: ependymal and choroid plexus epithelial cells are carrying, unexpectedly, fewer and shorter cilia, are poorly differentiated, and the mice develop hydrocephalus. TRIP6 carries numerous protein interaction domains and its functions require homodimerization. Indeed, TRIP6 disruption in vitro (in a choroid plexus epithelial cell line), via RNAi or inhibition of its homodimerization, confirms its function in ciliogenesis. Using super-resolution microscopy, we demonstrate TRIP6 localization at the pericentriolar material and along the ciliary axoneme. The requirement for homodimerization which doubles its interaction sites, its punctate localization along the axoneme, and its co-localization with other cilia components suggest a scaffold/co-transporter function for TRIP6 in cilia. Thus, this work uncovers an essential role of a LIM-domain protein assembly factor in mammalian ciliogenesis.

## Abstract (web summary)

Cilia, tiny outgrowths of cells, are essential for life. Here, the author’s describe the scaffold protein TRIP6, which promotes the assembly of ciliary proteins required for ciliogenesis, and show that its absence results in hydrocephalus.

## Introduction

Cellular interactions, via adhesion between cells or between cells and extracellular matrix, are hallmarks of multicellular organisms, and are formed by multiprotein complexes at cellular membranes. Their nature is transient in that adhesions are disassembled and reformed during diverse processes such as cellular migration, cell division, and tumorigenic transformation. Communication of neighboring cells, also of different cell types, is also mediated by specialized cellular interaction complexes, for example gap junctions. Development and function of the brain are prime examples of such cell–cell communications^1^, from adherens junctions^2,3^ and gap junctions to polarity regulation, actin-mediated motility^4^, and cell–matrix interactions^5^.

The formation of multiprotein complexes, including those involved in adhesions, often requires assembly factors. A family of scaffold proteins, carrying several protein interaction domains, serves as platform for protein–protein interactions: the seven-member ZYXIN LIM domain family^6^ including ZYXIN, lipoma-preferred partner LPP, AJUBA, WNT interacting protein WTIP, LIMD1, MIGFILIN, and thyroid hormone receptor interacting protein (TRIP6). Most of these proteins localize at focal adhesions and adherens junctions, compatible with their reported role in the assembly of adhesion complexes (as first described to be localized in cell–matrix adhesions by Beckerle^7^). Their interaction partners and the cellular functions of these complexes, from actin organization/cell migration^8–10^ through tension sensing^11–13^ to signal transduction^14,15^, have been elaborated in numerous cell culture experiments. The ZYXIN family members show significant structural and functional similarities^6^, and exhibit widely similar tissue specific expression pattern. Indeed, most of them seem to fully substitute for each other, as indicated by the viability and—by and large—normal adult life of mice with deletion of the genes for ZYXIN^16^, LPP^17^, AJUBA^18^, LIMD1^19^, and MIGFILIN^20^.

In contrast, in embryonic and early postnatal brain only two members of the ZYXIN family—*ajuba* and *trip6*—are expressed, and in a non-overlapping spatio-temporal pattern. *Ajuba* expressing cells were observed in early embryogenesis (embryonic days E10 to E13) and postnatally (mouse genome informatics atlas, ref. ^21^). *Trip6* was detected from E14.5 to early postnatal days with its expression restricted to the SOX2 positive cells of the ventricles^22^, and were reported to be expressed, also transiently, in the embryonic hippocampus, in the medial habenular nucleus, in the ventral posterior complex and in the thalamus^23^. In the adult mouse, single cell RNA profiles from the ventricular zone (VZ) identified ependymal cells expressing *trip6*, but also *zyxin* and *ajuba*^24^. Such analyses have not yet been performed for the embryonic VZ.

Here, we show that TRIP6 expression in the brain is spanning the embryonic (E14.5) to early postnatal period. This brain-restricted and exclusive expression pattern, solely of TRIP6 among all ZYXIN family proteins, encouraged us to delete the gene in the mouse. While the mice were viable—similarly to mice with deletion of genes encoding the other ZYXIN family members—we observed a major phenotype: development of an obstructive hydrocephalus. Investigation into the underlying etiology led us to the discovery of an unexpected function of TRIP6 in brain ciliogenesis.

## Results

### TRIP6 expression in the brain

**Fig. 1:**
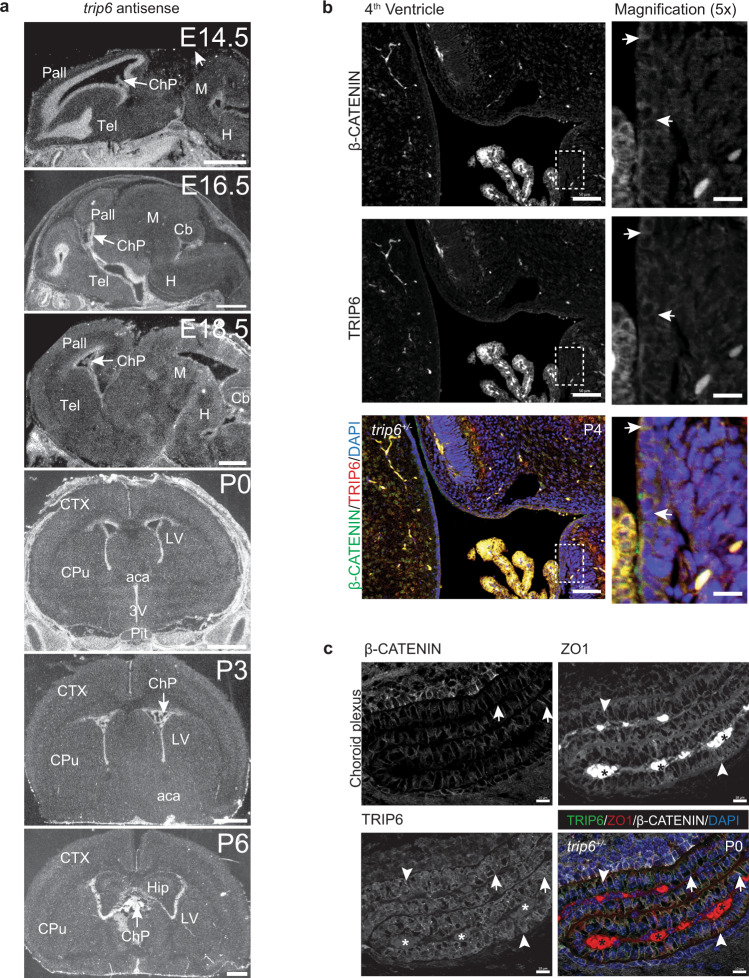
Expression of *trip6* in embryonic and postnatal brain. **a** mRNA expression in *trip6*^*+/+*^ mouse brain sections by in situ hybridization (ISH) depicting embryo sagittal sections (E14.5–E18.5) and postnatal coronal sections (P0–P6). Pall pallium, M midbrain, Tel telencephalic vesicle, H hindbrain, ChP choroid plexus, Cb cerebellum, CTX cerebral cortex, CPu Caudate Putamen, aca anterior commissure, Pit pituitary, LV lateral ventricle, 3V third ventricle, Hip hippocampal formation. Choroid plexus locations within the lumen of the ventricles are indicated by arrows. Control sense ISH is shown in Supplementary Fig. 1a, b. Immunofluorescence labeling of TRIP6 in ependymal cells lining the 4th ventricle (**b**) and choroid plexus epithelium (**c**) demonstrates its co-localization with β-catenin (arrow) and ZO1 (arrowhead). Nuclei were stained with DAPI. The marked areas are magnified in the insets. Ages are indicated in the figure. For number of mice analyzed, please see “Methods” section. Asterisk (*) indicates the autofluorescence of the choroid plexus vasculature. Scale bars: 1 mm (**a**), 50 μm and 250 μm (**b **and magnified images, respectively), 10 μm (**c**).

We confirmed *trip6* expression in the murine brain by radioactive in situ hybridization (ISH) (Fig. 1a). Widespread and intense *trip6* hybridization signals were observed in the extracranial somatic tissues (for whole skull sections see Supplementary Fig. 1a, b). In the embryonic central nervous system (CNS) however, *trip6* expression was highly restricted and limited to areas bordering the ventricles (Fig. 1a, E14.5–E18.5, sagittal sections), in agreement with published data^22^. A similar *trip6* expression pattern persisted after birth (Fig. 1a, postnatal days P0–P6, coronal sections), albeit at reduced expression level, and was undetectable by ISH beyond P10. *Trip6* mRNA expression was also detected in choroid plexus, e.g., at E14.5, E18.5, P0, and P3 (Fig. 1a, arrows). Immunostaining of the protein confirmed the ISH data and identified the exact region and cell type expressing TRIP6: a thin layer of cells bordering the ventricles in the VZ—likely ependymal cells—and epithelial cells in the choroid plexus anlage, both during embryogenesis (Supplementary Fig. 1c) and postnatally (Fig. 1b).

As expected for a member of the ZYXIN family, TRIP6 co-localized with β-catenin (Fig. 1b, c and Supplementary Fig. 1c), ZO1 (Fig. 1c) and N-cadherin (Supplementary Fig. 1c), marking predominantly the cellular membranes of both ependymal cells and choroid plexus epithelium. Oblique sections through the embryonic VZ displayed the net-like structure of the cell adhesions co-labeled by anti-β-catenin, N-cadherin, and TRIP6 antibodies (Supplementary Fig. 1c). Absence of TRIP6 did not alter the β-catenin and ZO1 localization (Supplementary Fig. 1d). Thus, TRIP6 is localized in cellular adhesion complexes, along the ventricle and in the choroid plexus epithelium.

### *Trip6* gene deletion in the mouse results in formation of a hydrocephalus

**Fig. 2:**
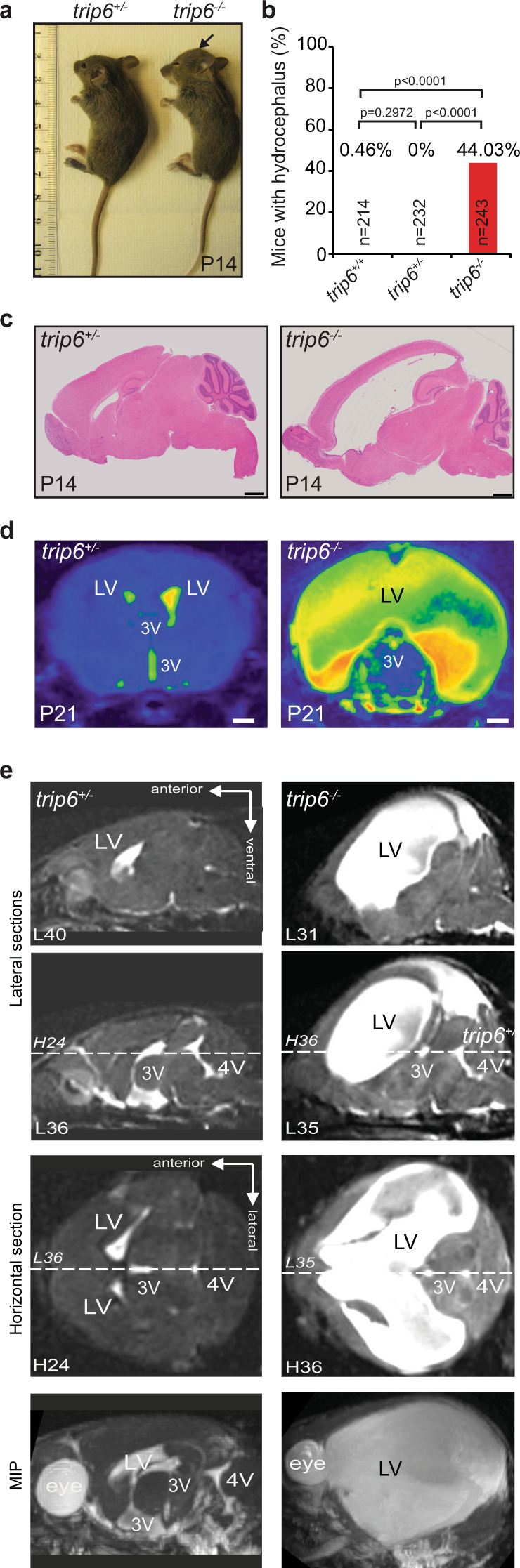
*trip6* deletion causes perinatal hydrocephalus. **a** Macroscopic view of cranial malformation indicative of hydrocephalus (arrow) in a *trip6*^−/−^ mouse (unaffected *trip6*^+/−^ littermate shown for comparison). **b** Quantification of hydrocephalus incidence in control and *trip6*^−/−^ mice. n number of mice assessed (*p* < 0.0001, two-tailed chi squared test; see also Methods and Supplementary Table 5). Source data are provided as a Source Data file. **c** H&E stained sagittal brain sections showing enlarged LVs in a *trip6*^−/−^ mouse (unaffected *trip6*^+/−^ littermate shown for comparison). **d** Coronal brain MRI images from control and hydrocephalic mice at the level of lateral (LV) and 3rd ventricle (3V) demonstrate the enlargement of LV, but not 3V, in the *trip6*^−/−^ mouse. In the colorized heat maps the fluid-filled ventricles appear yellow. **e** Lateral (“sagittal”) (L) and horizontal (H) brain MRI sections and lateral view of a maximum intensity projection (MIP) reconstruction depicting the ventricular system (white) in mice of the indicated genotypes (P21). Dashed lines (L and H) indicate the section planes of lateral and horizontal views, respectively. The LV is seen only in L40 and not in L36 in the wt. In control, LV, vs. 3V and the 4th ventricle (4V) can only be visualized in separate lateral sections (L40, L36) while the LV of *trip6*^−/−^ encompasses several sections (L31, L35). In contrast to LV, the 3V and 4V are not enlarged. The MIP views show the entire ventricular system. Note the massive expansion of the ventricular system in *trip6*^−/−^ compared to the heterozygote. Scale bar: 1 mm (**c**, **d**). For 3D reconstruction of the complete set of sections, please see Supplementary Movies 1 and 2.

The generation of knock-out (*trip6*^−/−^) mice by standard technology is outlined in Supplementary Fig. 2. Deletion of *trip6* was confirmed at mRNA level, in choroid plexus, via RT-qPCR (Supplementary Fig. 2d) and at protein level, in several organs, via immunoblotting (Supplementary Fig. 2e). The *trip6*^*−/−*^ mice were viable through birth, but a large fraction (44%) in the C57BL/6J background developed a severe phenotype: hydrocephalus (Fig. 2a, b). Haematoxilin-Eosin (H&E) stained brain tissue revealed significant extension of the lateral ventricles (LV) in these animals (Fig. 2c). In the fraction of *trip6*^−/−^ mice escaping the formation of hydrocephalus, as well as in heterozygotes, the overall brain morphology was not altered.

High resolution magnetic resonance imaging (MRI) showed that the LVs were significantly enlarged, whereas no ventriculomegaly was detected in the 4th ventricle (Fig. 2d, e). In serial sagittal and horizontal MRI sections, as well as in maximum intensity projection (MIP) reconstructions of P21 brains the 3rd ventricle was in part concealed by the large LV expansion (Fig. 2d, e and Supplementary Movies 1 and 2). From histological sections at earlier postnatal days the 3rd ventricle appeared unaffected (see also below). LV enlargement was first detected at P1 and maximal extension was reached at P24. This phenotype speaks for a cerebrospinal fluid (CSF) circulation block in the foramen of Monro (upstream of the 3rd ventricle) and/or in the aqueduct of Sylvius, between 3rd and 4th ventricle (reviewed in ref. ^25^)—suggesting the existence of an obstructive hydrocephalus.

A small cohort of 22 *trip6*^*−/−*^ mice, that had not developed a hydrocephalus perinatally, were followed for up to 400 days. They did not form a hydrocephalus later during the observation time, and their life span did not appear to be shortened in comparison to *trip6*^*+/+*^.

As congenital hydrocephalus is often accompanied by delamination of ependymal cells from the dorsal wall of the aqueduct, with fusion of the ventral and dorsal walls and consequently aqueduct obliteration^26,27^, we focused on analysis of the ependymal cell layer.

### Defective ependymal cells in *trip6*^*−/−*^ brain

**Fig. 3:**
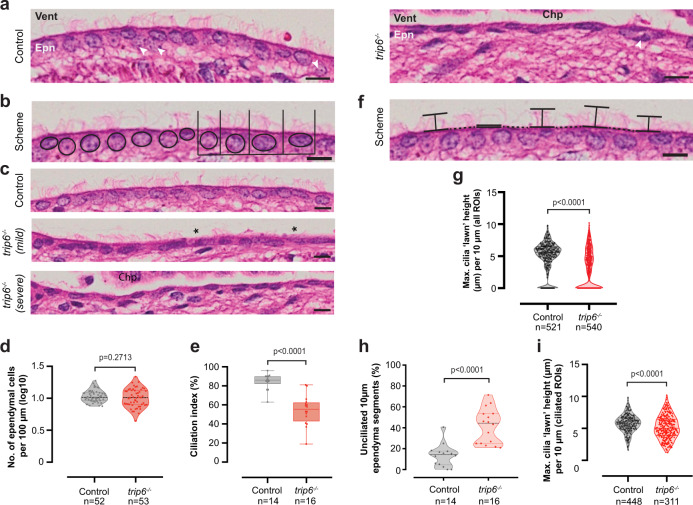
Histological characterization and quantification of periventricular ependymal cells and their cilia. **a** Representative images of H&E stained ependyma, comprising ependymal cells (Epn) and interspersed basal cells (arrowheads), lining the 4th ventricle (Vent) in *trip6*^*+/−*^ (Control) and *trip6*^*−/−*^ mice. Note the poorly differentiated epithelial layer comprising atrophic ependymal cells that exhibit “endothelial” (flattened) cell morphology and aberrant ciliation, as well as the relative sparsity of basal cells in the *trip6*^*−/−*^ brain. **b** Semi-schematic depiction of the segmentation method used in cell number and ciliation index quantification of the ependyma. Individual ependymal cells were determined by their nuclei (circles) and inspected for the presence of cilia on their apical site (lines). **c** Representative images depicting native, mild and severe phenotypes for cell number and ciliation of control and *trip6*^*−/−*^ mice. Quantification demonstrated that although the mean number of ependymal cells was not altered (**d**), ciliation is significantly affected (**e**). [The box plot (**e**) indicates the median (middle line), the interquartile range from 1st to the 3rd quartile (box) and the range of the minima to maxima (whiskers)]. **f** Semi-schematic depiction of the method used for quantification of cilia length and coverage of the ependyma. Individual segments of 10 µm were determined (horizontal full and dotted lines) and maximum cilia “lawn” height was measured at each second segment (vertical lines) for quantification and found to be reduced in *trip6*^*−/−*^ mice (**g**). The coverage of cilia (indicated as the fraction of unciliated segments per 100 µm) is significantly reduced in *trip6*^*−/−*^ mice (**h**). The length of cilia (determined as maximum cilia “lawn” height per 10 µm ciliated segment) was also significantly reduced (**i**). Further information on quantification methodology and statistical tests performed can be found in “Methods” section (including Supplementary Table 7). The statistical significance of the analyses is represented by the *p*-values indicated in the graphs (**e**, **d**, **g**, **b**, **h**, **i**). Source data are provided as a Source Data file. Chp choroid plexus. Asterisk (*) indicates unciliated areas. Scale bars: 10 µm.

The ventricles of early postnatal control (trip6^***+/+***^) brain (Fig. 3a) are lined by a single layer of multiciliated cuboidal-to-columnar epithelial cells—the ependymal cells—comprising the ependyma. The ependymal layer is interspersed with basal cells some of which are stem cells (Fig. 3a, arrowheads), providing a neurogenic niche^28^. Analysis of *trip6*^−/−^ brain sections (Fig. 3a) revealed a poorly differentiated epithelial layer comprising atrophic ependymal cells that exhibit “endothelial” aspects (i.e., flattened cell morphology and reduced cytoplasm, as indicated by high nuclear/cytoplasmic ratio) as well as aberrant ciliation demonstrated by defects in cilia number and length (see below). Furthermore, only few basal cells were observed (Fig. 3a), suggestive of defective cell renewal, or reflecting an early cause of poor ependymal differentiation. Examples of *trip6*^−/−^ ependymal layers are shown in Fig. 3c indicating different degrees of poor differentiation.

**Fig. 4:**
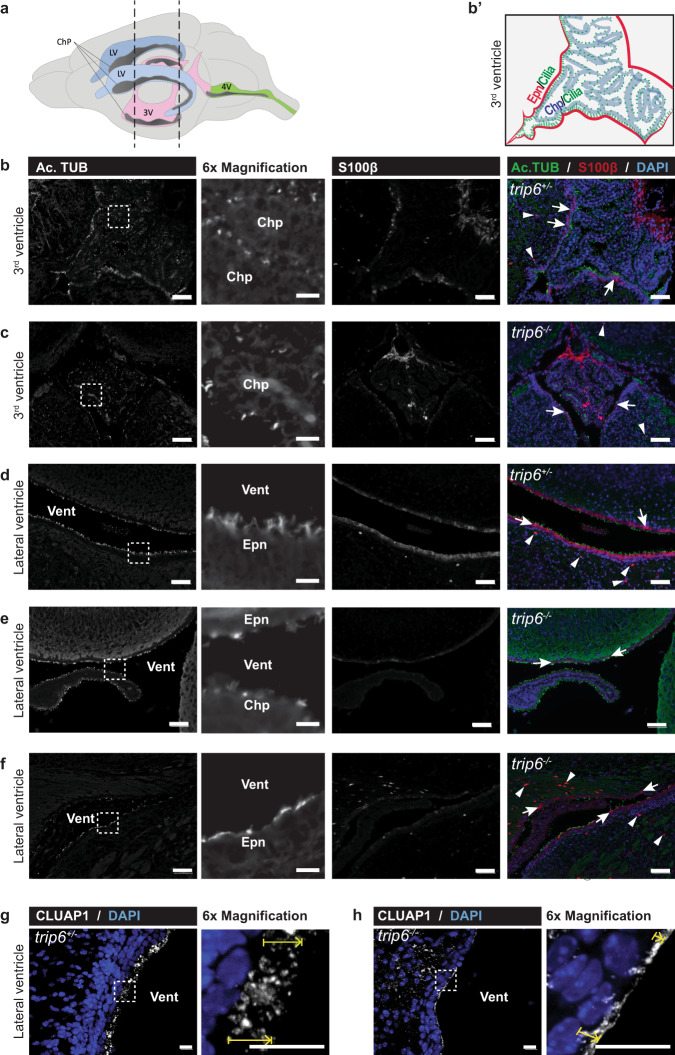
Defective differentiation of ependyma and choroid plexus in *trip6*^−/−^ mice. Schematic illustration of mouse brain ventricles and choroid plexus, with vertical lines indicating the approximate coronal section planes analyzed (**a**). Scheme (**b′**) and immunofluorescence microscopy analysis (**b**) of ependyma (Epn) and choroid plexus (Chp) within the 3rd ventricle (Vent: ventricular lumen) in control brain sections, illustrating ciliation and ependymal cell differentiation [visualized by acetylated tubulin (Ac.TUB) and S100β labeling, correspondingly]. Nuclear DNA is stained by DAPI. Note that S100β is expressed in ependymal (arrows, **b**–**f**) but also in non-ependymal cells (arrowheads, **b**–**f**). Trip6 deletion (**c**) results in differentiation defects indicated by reduction in ciliation and S100β expression. Importantly, these defects [which are apparent also in the LV (Vent) (**e**, **f** *cf*. control **d**)] are observed in mice with (**e**) or without (**f**) hydrocephalus, suggesting that differentiation defects precede development of this pathology. For higher magnification images, please see Supplementary Fig. 3. Defective ciliation in *trip6*^−/−^ brain is further demonstrated by labeling the ciliary transport protein CLUAP1 (**h**). Yellow arrows indicate the height of the cilia lawn in control (**g**) and *trip6*^−/−^ (**h**) specimens. Scale bars: 50 μm (**b**–**f**), 10 μm (**g**, **h**).

Immunofluorescence labeling, using anti-S100β, confirmed the defective differentiation of ependymal cells in *trip6*^−/−^ brain (Fig. 4 and Supplementary Figs. 3 and  4a; ependymal cells indicated by arrows). Note that S100β is expressed not exclusively in ependymal cells, but also in some glia cells (Fig. 4 and Supplementary Fig. 3, arrowheads; Supplementary Fig. 4a, stars) in agreement with previous reports^29^. In addition to this defective morphology, elimination of S100β-expressing cells from the VZ was occasionally observed (Supplementary Figs. 3 and 4).

The “endothelial” appearance of the ependymal layer is unlikely to be caused by the hydrocephalic pressure increase, because a similar thinning of ependyma and choroid plexus was also observed in the 3rd ventricle (Fig. 4b, c) and in the 4th ventricle (Supplementary Fig. 4a), which do not present with enlargement, consistent with the fact that the 3rd and 4th ventricles are downstream of the CSF circulation block.

To rule out that the atrophic “endothelial” cell morphology of the ependymal layer and the decreased expression of S100β was due to delamination/cell loss, we quantified the number of ependymal cells in H&E-stained sections of the 4th ventricle (as described under see “Methods” section, and exemplified in Fig. 3b). Despite the wider variability (*f*-test, *p* = 0.0026) in cell number per segment of ependyma analyzed, in *trip6*^*−/−*^ brain, there is no statistically significant difference in the mean ependymal cell density between control and *trip6*^*−/−*^ brain (Fig. 3d). Thus, at least in the *trip6*^*−/−*^ 4th ventricle, there was no overall reduction in ependymal cells.

**Fig. 5:**
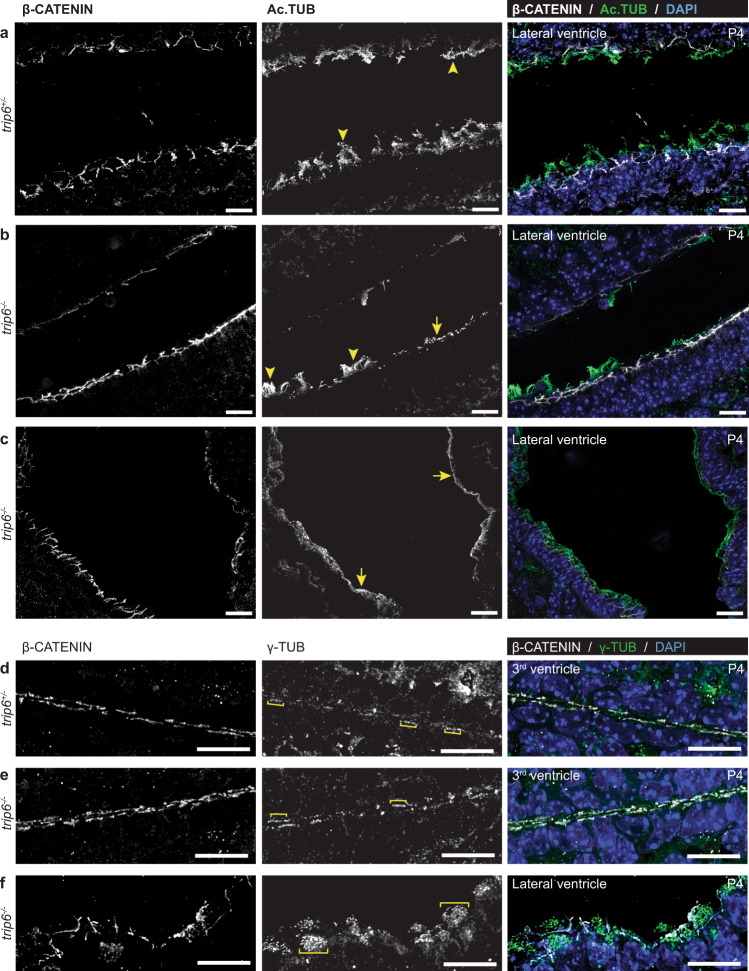
Defective ciliation of ependyma in *trip6*^−/−^ mice. Deconvolved immunofluorescence microscopy images of the ependyma of three *trip6*^−/−^ and two control *trip6*^+/−^ mice (all at P4) depict, in high spatial resolution, adhesions (labeled by an anti-β-catenin antibody), cilia (anti-acetylated α-tubulin labeling), and basal bodies (anti-γ-tubulin labeling). An animated 3-D reconstruction of the images is presented in Supplementary Movies 3, 4, and 5. There are no obvious adhesion defects in the ependyma, neither in the LV (**a** c*f*. **b**, **c**, **f**) nor in the 3^rd^ ventricle (**d** *cf*. **e**), as demonstrated by comparison of control (**a**, **d**) with *trip6*^−/−^ (**b, c**, **e**, **f**) specimens (β-CATENIN). In contrast, in the LV, defective ciliogenesis in *trip6*^−/−^ mice of mild (**b**) or severe (**c**) phenotype (degree of cilia absence) is demonstrated by ependyma areas presenting with a thin layer of acetylated α-tubulin labeling (Ac.TUB, arrows). For comparison, normal cilia bundles are indicated by arrowheads, in control (**a**) and *trip6*^−/−^ (**b**, **c**) specimens. (see also Fig. 7d*cf*. e, for higher resolution images of cilia). Regular formation and ventricular localization of basal bodies, in *trip6*^*−/−*^ ependymal cells (**e**, **f**) of 3rd and LVs, is visualized by the punctate γ-tubulin staining pattern. The brackets indicate clusters of basal bodies within single ependymal cells (γ-TUB; **d**–**f**). Scale bars: 10 μm.

One of the essential functions of ependymal cells is the formation of motile cilia. We quantified different aspects of ependymal ciliation, in the 4^th^ ventricle (Fig. 3, see “Methods” section for detailed description and statistical analysis). The aberrant ciliation of the ependyma, observed by histopathological analysis, was confirmed by this quantification, in H&E-stained brain sections (Fig. 3c), and by cilia-specific immunofluorescence (Figs. 4 and 5 and Supplementary Movies 3 and 4).

First, the ciliation index (defined as the percentage of ciliated ependymal cells per ventricular section analyzed) was found to be significantly reduced—by 30%—in *trip6*^−/−^ (Fig. 3e). Second, we quantified cilia length. Briefly, ependyma segments of 10 μm in length, along the ventricular wall, were examined for the presence of cilia, and cilia length was subsequently measured (Fig. 3f). The percentage of un-ciliated segments of ependyma (marked by * in Fig. 3c) was very significantly increased (3.4 times) in *trip6*^*−/−*^ (Fig. 3h). Moreover, even in the ciliated segments, the cilia “lawn” height (defined as an approximate measure of cilia length) was significantly reduced (Fig. 3i), with median cilia length being reduced by ca. 14% (Fig. 3i). Figure 3g summarized the cilia height through all 10 μm segments (the data of 3h and 3i combined). These results demonstrate that the ciliation index likely underestimates the phenotype, which is better represented by analysis of ciliation along ependyma segments (Fig. 3g), rather than per ependymal cell (Fig. 3e).

Immunofluorescence microscopy with cilia-specific markers confirmed the cilia defect in *trip6*^*−/−*^ ventricles. Ependymal cell layers labeled with antibodies against acetylated α-tubulin (Ac.TUB) (cilia marker^30^) showed clusters of cilia at the *trip6*^+/−^ ependyma that were reduced or eliminated in *trip6*^−/−^ (Fig. 4d, e; compare *trip6*^*−/−*^ in e with *trip6*^+/−^ in d; and compare Supplementary Fig. 3b, c with a). The ependymal phenotype was identical in the LVs of hydrocephalic (Fig. 4e) and non-hydrocephalic (Fig. 4f) *trip6*^−/−^ mice, indicating that it was not caused by increased fluid pressure. Immunofluorescence microscopy using another cilia marker (clusterin-associated protein1; CLUAP1/IFT38) revealed, similarly, the diminished cilia length (Fig. 4g, h).

To improve spatial resolution of subcellular structures, including the cilia, Z-stacked optical sections of immunofluorescence microscopy images were deconvolved and 3D reconstructed (Fig. 5 and Supplementary Movies 3, 4). Regions of the *trip6*^*−/−*^ LV confirmed the greatly reduced cilia formation or cilia elongation defects (arrows, Fig. 5b, c). Nevertheless, in agreement with the quantification data (Fig. 3) ependyma regions with apparently normal cilia were also observed (arrowheads; Fig. 5, compare a with b; Supplementary Movies 3 and 4). Reduction in S100β intensity correlates with aberrant/absent ciliation (Supplementary Fig. 4a, b) suggestive of a link between S100β acquisition and cilia formation.

Notably, the formation and subcellular localization of basal bodies appeared unaffected by *trip6* deletion (Fig. 5e, f compare to 5d; Supplementary Movie 5). Taken together these data suggest that cilia elongation, rather than nucleation, is compromised by the *trip6* deletion.

Deletion of *trip6* also affected the choroid plexus, both in size (Supplementary Fig. 1d) and in ciliation (Fig. 4b, c). As ependymal and choroid plexus cilia differ both in developmental fate and in function (see “Discussion” section), this observation suggests that absence of TRIP6 affects cilia more generally.

### Trip6 deletion does not affect adherens junctions

**Fig. 6:**
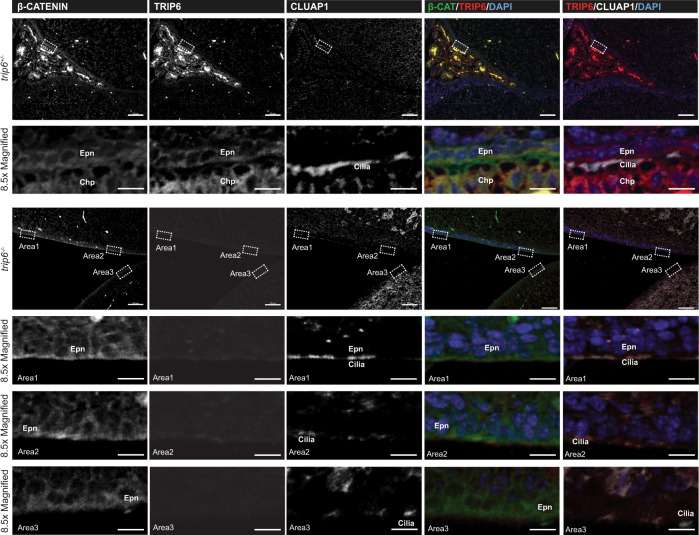
*Trip6* deletion affects ciliogenesis but not cell-cell adhesion. Immunofluorescence microscopy-based expression and localization analysis of cilia (CLUAP1) and cell-cell adhesion (β-CATENIN) markers, as well as of TRIP6, in the LV of control (*trip6*^+/−^) and *trip6*^−/−^ mouse brain. Their comparison demonstrates TRIP6 deletion and ciliation defects in *trip6*^−/−^ specimens, but no obvious adhesion defects. Magnification panels depict selected areas (indicated by white frames in the low magnification images), re-oriented with the ventricular lumen positioned at the lower part of each image. Epn ependyma, Chp choroid plexus. Scale bar: 50 μm.

In control brain, TRIP6 co-localized with β-catenin and N-cadherin (Figs. 1b and 6 and Supplementary Fig. 1c, d) as well as with ZO1 (Fig. 1c). This was to be expected given the role of TRIP6 in the assembly of adhesion complexes in cell culture^8,9,13^. The role of adhesion complexes in ciliogenesis is well documented^31^. Thus, in view of the ciliary malformation of choroid plexus and ependymal cells in *trip6*^−/−^ mice, we asked whether the adhesions were affected. Surprisingly, localization of β-catenin and ZO1 at cellular membranes was not altered in *trip6*^−/−^ mice (Figs. 5 and 6 and Supplementary Fig. 1d, Supplementary Movies 3, 4) indicating that cell-to-cell adhesions and tight junctions do not—at least not structurally—depend on TRIP6. Despite maintenance of β-catenin labeled cellular adhesions and recruitment of CLUAP1 to the ventricular surface of the *trip6*^*−/−*^ ependymal cells, cilia failed to elongate (Fig. 6, several areas are selected and magnified for better visualization).

### TRIP6 is associated with ciliary structures

So far we have documented that ependymal and choroid plexus are malformed in the absence of TRIP6 and that they lack the normal abundance of cilia. At which level does TRIP6 act?

**Fig. 7:**
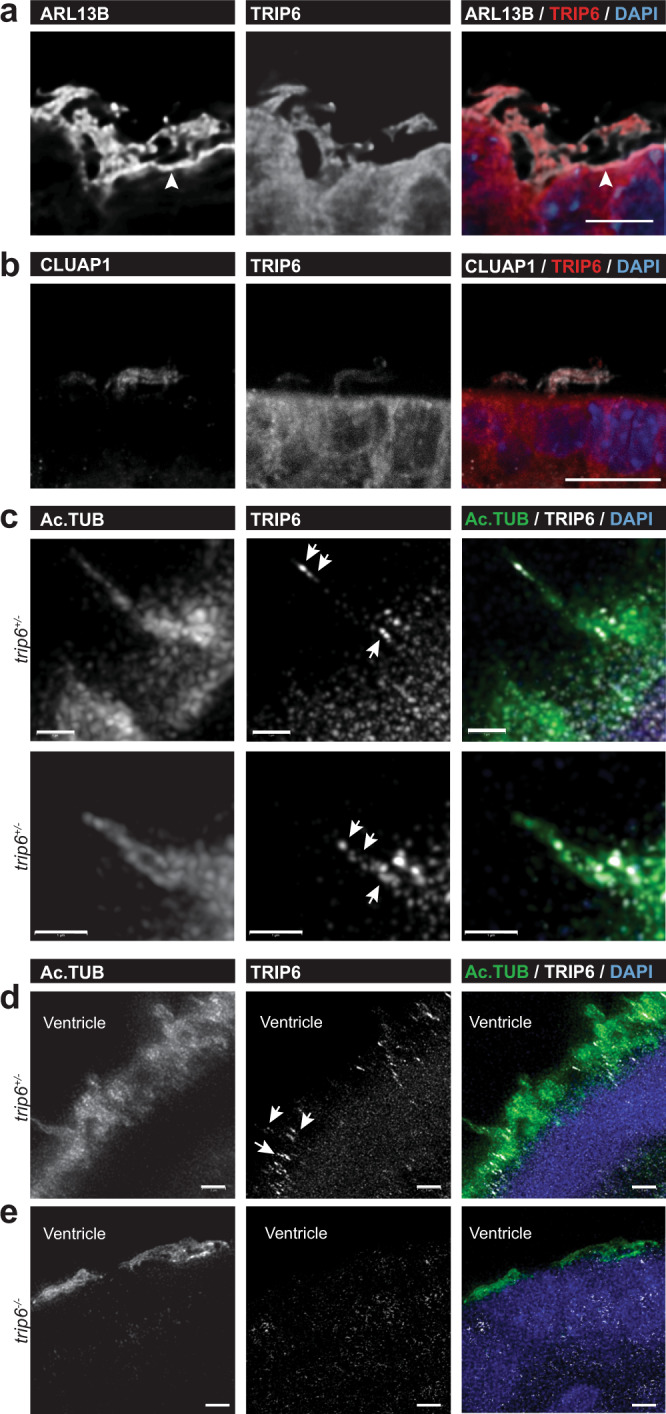
Localization of TRIP6 in motile cilia of brain ependyma, via super resolution microscopy. Co-localization of TRIP6 with the cilia markers ARL13B (**a**) and CLUAP1 (**b**) in cilia of control mice ependyma, via double-labeling immunofluorescence microscopy (Arrowhead indicates localization of ARL13B also at the ventricular membrane, see Results). Super resolution microscopy (**c**–**e**) further demonstrates the co-localization of TRIP6 with acetylated α-tubulin (Ac.TUB) within individual cilia (**c**). In controls (**c**, **d**), note the punctate labeling of TRIP6 (arrows) revealed by super resolution microscopy, in contrast to the more uniform Ac.TUB labeling pattern along the axoneme (**c**, **d**). In *trip6*^−/−^ ependyma (**e**), Ac. TUB has accumulated along the ventricular cell surface but the axoneme failed to elongate. Scale bars: 5 μm (**a**), 10 μm (**b**), 1 μm (**c**), 2 μm (**d**, **e**).

The decisive observation was obtained by co-immunofluorescence microscopy: ARL13B (the cilium component ADP ribosylation factor like GTPase 13B^32,33^; Fig. 7a), CLUAP1 (Fig. 7b) and TRIP6 co-stained cilia clusters in brain sections from P4 *trip6*^*+/−*^ mice (Fig. 7a, b). These data suggest that TRIP6 is part of the ciliary structure. A more detailed localization of TRIP6 was obtained by super resolution microscopy. TRIP6 ‘puncta’ were detected at the base, along the length and at the tip of the cilium of LV ependymal cells (Fig. 7c, d). The distribution of TRIP6 staining differed among cilia, possibly capturing different subciliary locations of TRIP6 in the process of intraflagellar transport. In the absence of TRIP6, Ac.TUB foci accumulated along the ventricular surface without proper cilia formation (Fig. 7e, compare to 7d; see also Supplementary Movie 4) suggesting that the ciliary axoneme failed to elongate.

### Requirement for TRIP6 in ciliogenesis

To further investigate the putative roles of TRIP6 in ciliogenesis, we used the murine choroid plexus cell line Z310^34^. In monolayer culture, these cells formed adherens junctions (Supplementary Fig. 5a) colocalizing β-catenin and TRIP6, similarly to choroid plexus epithelial cells in vivo (Fig. 1c). In agreement with published reports^9^, TRIP6 also localized at focal adhesions as illustrated by anti-paxillin immunofluorescence staining (Supplementary Fig. 5b, d).

**Fig. 8:**
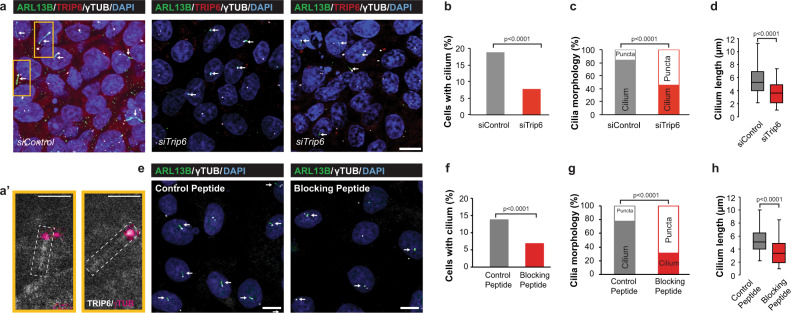
Downregulation of *trip6* by siRNA or blocking of TRIP6 dimerization, in the choroid plexus-derived cell line Z310, impairs cilium elongation or ciliogenesis. Z310 cells, transfected with the indicated siRNAs (siTrip6 or siControl) (**a**) or peptides (dimerization blocking or control) (**e**), were serum-starved to induce primary cilium formation (see “Methods” section for detailed description). The cells were analysed by immunofluorescence microscopy to visualize basal bodies (labeled by anti-γ-tubulin antibody) and the primary cilium (anti-ARL13B), and were counterstained with anti-TRIP6 and DAPI (**a’** shows magnified cilia from panel **a**). Both inhibitory treatments significantly impaired primary cilium formation (**b**, **f**; analysed by two-tailed Fisher’s exact test) or—in those cells carrying cilia-like structures—cilium morphology (**c**, **g**; two-tailed Fisher’s exact test) and length (**d, h**; two-tailed Student’s *t*-test). Both phenotypes were adversely and significantly affected. The box plots (**d**, **h**) indicate the median (middle line), the interquartile range (IQR) from 1st to the 3rd quartile (box), and the upper edge (Q1 + 1.5*IQR) to lower edge (Q1 − 1.5*IQR) (whiskers). In the morphological assessment, “puncta” indicate basal bodies without associated axoneme. For detailed description of quantification and statistics, see “Methods” section. Source data are provided as a Source Data file. Additional analysis of the RNAi experiments is presented in Supplementary Fig. 5. Scale bars: 10 μm (a, a', e).

Upon serum starvation, Z310 cells developed primary cilia. In the cells treated with siControl, TRIP6 localized at the axoneme, best seen in the magnification of two individual cilia (Fig. 8a’). Is TRIP6 required for the formation of primary cilia in vitro? Treatment of Z310 cells with *trip6* siRNA (*siTrip6*) resulted in reduced number (Fig. 8a–c) and length (Fig. 8d) of ciliary structures compared to cells transfected with non-targeting siRNA (siControl). Most cells transfected with *siTrip6* produced, *in lieu* of a primary cilium, only a single “punctum” comprising basal body (labeled with anti-γ-tubulin (γTUB)) and a very short axoneme (marked by ARL13B). The increased number of puncta in cells with downregulated *trip6* suggests that cilia initiation is reduced or delayed in the absence of TRIP6.

To better understand how is ciliogenesis compromised, we analyzed the localization of several ciliary components at 24 h post cilium induction. While puncta in the control culture carried acTUB, γTUB, IFT88, CLUAP in addition to ARL13B, as expected from an initiating cilium, the puncta observed in *siTrip6* treated cultures differed from those in the control: Several ARL13B and γTUB positive puncta lacked acTUB, IFT88, or CLUAP (Supplementary Fig. 6b). Moreover, γTUB staining was often reduced. These conditions resemble an arrest very early in cilia initiation^35^, also compatible with the reported colocalization of TRIP6 with centriolar appendices^36^. Other puncta presented with excessive accumulation of acTUB or CLUAP around the basal body (Supplementary Fig. 6c). Finally, abnormal cilia with proximal or distal accumulation of ciliary components were observed (Supplementary Fig. 6d). Similar arrest phenotypes have been reported upon deletion of individual intraflagellar transport (IFT) components^37^.

Quantification confirmed that *siTrip6* significantly reduced the number of cells carrying cilia (Fig. 8b). The fraction of cells with properly formed cilia was diminished (Fig. 8c) and—in those cells that formed cilia—the cilium length was very significantly reduced (Fig. 8d). Thus, in addition to its role in motile cilia formation, TRIP6 is obviously required for primary cilium formation and elongation.

In contrast to ciliogenesis, downregulation of *trip6* in Z310 cells by siRNA did not abolish either adherens junctions or focal adhesions (Supplementary Fig. 5d, e), as had been observed in other cell types (Figure EV4 in ref. ^13^). At focal adhesions, residual TRIP6 was still visible indicating a longer protein half-life. Most likely, TRIP6 is stabilized when incorporated into focal adhesions, remaining present longer than at adherens junctions. Thus, in cultured cells as in the brain, downregulation of TRIP6 did not affect adhesion complexes (compare with Figs. 5 and 6).

TRIP6 carries three C-terminal LIM domains with different interaction specificities. Moreover, the N-terminal half harbors additional motifs for binding proteins. The ability to act as an assembly platform is further increased by two dimerization domains^38^. To examine whether dimerization was required for the ciliogenetic role of TRIP6, we blocked the former by overexpressing a peptide competing with the dimerization domains in Z310 cells. The resulting phenotype was similar to that obtained after downregulation of *trip6* expression by siRNA: the number and length of the cilia were significantly reduced upon transfection with the blocking peptide as compared to transfection with a control scrambled version (Fig. 8e–g). Additionally, the block of dimerization caused “puncta” appearance of severely reduced cilia length (Fig. 8h). These data suggest that TRIP6 requires the full complement of protein binding options, and that it may serve as an assembly factor for multiprotein complexes involved in cilium formation. Hence, in addition to its role in motile cilia formation, TRIP6 is obviously required for primary cilium formation and elongation.

**Fig. 9:**
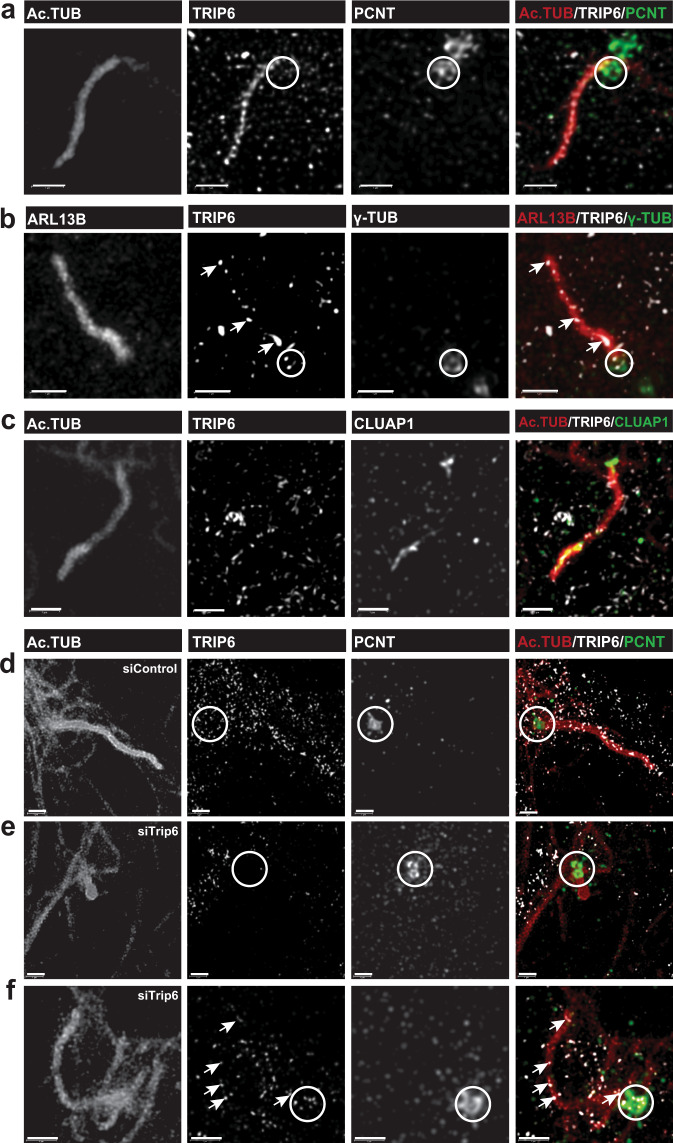
Localization of TRIP6 in primary cilia of choroid plexus-derived Z310 cells, via super resolution microscopy. Representative examples of six primary cilia, labeled with the structural cilia components acetylated α-tubulin (Ac.TUB), ARL13B or CLUAP, and with anti-TRIP6. TRIP6 localized in foci along the axoneme, exhibiting variable distribution among individual cilia (arrows in **b**, **f**) when compared to the more uniform sub-ciliary localization of Ac.TUB and ARL13B (**a**, **b**). Super resolution identifies two major axonemal sites of CLUAP (**c**). Additionally, TRIP6 partially co-localized with the basal body markers pericentrin (PCNT; **a**, **d**) and γ-tubulin (γ-TUB; **b**). Basal bodies are indicated by circles. Downregulation of *trip6* expression, by siRNA, reduced the length of the axoneme without affecting the basal body (**e** *cf*. **d**). Nevertheless, those cells presenting with elongated cilia, in the siTrip6-treated cultures (partial downregulation), were found to still express TRIP6 that localized along the axoneme (arrows, **f**) and in the basal body (circle, **f**). Scale bars: 1 μm.

Together, the in vitro phenotypes resulting from TRIP6 disruption corroborate our in vivo observation from *trip6*^*−/−*^ mice. Deletion, downregulation or inhibition of dimerization of TRIP6 leads to reduction in the percentage of ciliated cells. Even when cilia are formed, they are shorter (Figs. 3 and 9). Ciliary components are nevertheless still recruited to the ventricular surface of the ependymal cells in vivo (Figs. 5a, b, 6, and 7d), also indicated by the “puncta” morphology in the cultured choroid plexus epithelial cells (Fig. 8).

Additionally, similarly to the brain cilia phenotype (Fig. 7c), super resolution microscopy of the cilia of Z310 cells (Fig. 9) revealed a punctate distribution of TRIP6 along the length of the ciliary axoneme, somewhat differing in distribution between individual cilia (arrows in Fig. 9b, that depicts representative examples of TRIP6 localization from 28 cilia analyzed). This is suggestive of “snapshots” illustrating different stages of intraflagellar transport. In contrast, the structural cilia components Ac.TUB and ARL13B, were more uniformly distributed along cilia. TRIP6 also co-localized with γ-tubulin at the basal bodies (Fig. 9b; basal bodies indicated by circles). Compared to TRIP6, CLUAP1 was predominantly located at the distal and proximal ends of the cilia (Fig. 9c), in agreement with previous reports^39^.

The *siTrip6* induced “puncta”, shown in Fig. 8, represent in fact short cilia as revealed by super resolution microscopy (Fig. 9e; a total of 20 *siControl* and 15 *siTrip6* cilia were imaged, a selection is presented). Pericentrin (PCNT, a component of the pericentriolar material surrounding the basal bodies) localization was not affected. Due to partial downregulation of *trip6*, cells with full-length cilia were detected in the same culture (Fig. 9f). They expressed TRIP6 and localized it along the axoneme and at the basal body (Fig. 9f) similarly to cells transfected with the control *siRNA* (Fig. 9d).

## Discussion

We report that the LIM domain protein TRIP6 promotes the differentiation of ependyma in the developing mouse brain. Notably, we have uncovered a role for TRIP6 in brain ciliogenesis.

In humans, ciliogenesis deficiencies cause numerous diseases—ciliopathies—which affect many organs including the brain^25,40^. The ciliopathy-like phenotype in the brain of *trip6* knockout mice, which includes the formation of a hydrocephalus, led us to the discovery of a role for TRIP6 in the formation of ependymal cilia. In the absence of TRIP6 fewer and shorter cilia are formed, with a fraction of ependymal cells carrying the basal bodies without extended axoneme.

TRIP6 is a member of an assembly factor family of LIM domain proteins. Given their overlapping molecular properties and structural similarities, it is plausible to hypothesize that other members of the family could also function in ciliogenesis. This seems to be the case: in the *Xenopus* embryo the knockdown of WTIP altered the location of basal bodies and reduced cilium length^41^. ZYXIN, LPP as well as TRIP6 were detected in the ciliary membrane-associated proteome of inner medullary collecting duct cells^42^. In the brain, however, TRIP6 is the sole member of the LIM domain family expressed during the generation and maturation of the ependymal cells and differentiation of choroid plexus (between E14.5 and birth, Fig. 1, and mouse genome informatics atlas, ref. ^21^). Hence, it is not substituted by other family members. Although mice with gene disruptions of individual members of the ZYXIN family are viable and showed no severe phenotype, including no hydrocephalus^16–20^, an analysis of ciliogenesis in these mice has not yet been published. Therefore it remains to be shown whether LIM domain proteins function redundantly in ciliogenesis.

The brain ciliopathy in *trip6*^*−/−*^ mice resembles those phenotypes generated by a large variety of gene mutations or altered expression of genes which ultimately affect ciliogenesis (a comprehensive list can be found in ref. ^25^). Most frequently the axoneme length is affected^43^. To cite a few examples: a splice donor site mutation in the gene for axonemal protein coiled-coil domain 39 was found to be the cause of the progressive hydrocephalus (*prh*) mouse phenotype^44^. The length of motile cilia, reduced in *prh* mice, is apparently critical for function. Disruption of the gene encoding the ciliary component ARL13B also results in shortened cilia^32,45^. Interestingly, ARL13B acts at two different subcellular locations exerting two functions which can be distinguished by mutation of the cilia targeting sequence: cilia length regulation requires ARL13B ciliary localization while Wnt signaling control is mediated by ARL13B from “outside the cilia”^33^. As a last example, both inhibition of or enhanced actin polymerization affect cilia disassembly or formation^46–48^.

The defects in differentiation and ciliogenesis in the developing brain of the *trip6*^*−/−*^ mice are associated with severe hydrocephalus. However, hydrocephalus was observed in only 44% of the knockout animals, whereas the impaired ciliogenesis of ependymal cells was fully penetrant. Also ciliation of the choroid plexus appeared to be affected. These observations clearly suggest that hydrocephalus is not a direct consequence of the loss of TRIP6, but rather a secondary effect. Furthermore, the enlargement was restricted to the LVs, suggesting an occlusion of CSF passage downstream of these ventricles. This phenotype strikingly resembles human genetically determined non-communicating (obstructive) hydrocephalus, a condition caused by occlusion of CSF passage, which represents the most frequent hydrocephalus etiology besides CSF overproduction^25^. Thus, it seems that the obstructive hydrocephalus observed upon deletion of TRIP6 is caused by a CSF circulation block in the foramen of Monro (upstream of the 3rd ventricle) and/or in the aqueduct of Sylvius, between the 3rd and 4th ventricle.

Given the reported role of TRIP6, based on in vitro experiments, in both focal adhesions and adherens junctions^8,9^, one would hypothesize that loss of TRIP6 promoted detachment of ependymal cells, ultimately occluding the aqueduct. Such a mechanism has indeed been reported in cases of congenital hydrocephalus^26,27,49,50^ as well as in animal models of defective cell adhesion^51–53^. In the *trip6*^*−/−*^ mouse, the ependymal epithelium indeed exhibits poor differentiation presenting with atrophic cells of endothelial morphology and reduced expression of S100β (Fig. 3 and Supplementary Fig. 4) that may delaminate and, in the narrow CSF canals, cause blocks by adhering to the opposite wall. However, the deletion of *trip6* did not seem to affect formation of adherens and tight junctions, whilst the number of ependymal cells was not, at least in the 4th ventricle, significantly reduced. Whether cell–cell adhesion strength is reduced by the defective differentiation and results in clotting at critical sites, remains a hypothesis.

Adhesion complexes exert regulatory functions through actin dynamics^8,9^. Notably, adhesion complexes such as those localized at focal adhesions are also found in basal bodies, thus linking cilia with the actin cytoskeleton^31^. Actin polymerization indeed affects primary cilium length and function^43,54^. Thus, a putative regulation of the function of TRIP6 in ciliogenesis exerted via the actin cytoskeleton cannot be ruled out at this time. However, the localization of TRIP6 in the axoneme, combined with the fact that, in the *trip6*^*−/−*^ mouse, basal body translocation to the ventricular side of the ependymal cells was not compromised, do not speak for an actin-mediated mechanism.

In addition to its function in ependymal cell cilia, TRIP6 is also required for the formation of primary cilia. Furthermore, our own preliminary data have shown that *trip6* deletion also affected the airway cilia, suggesting that the protein is required for ciliogenesis in general. A role for TRIP6 in formation of the primary cilium (our in vitro analysis), of the ependymal cell motile cilia, of the airway motile cilia and of the choroid plexus cilia (our in vivo analysis) suggest involvement of the protein in a process shared by these distinct cilia types.

Primary and motile cilia differ in their structure and function (reviewed in refs. ^55,56^). Structurally, they differ in the central pair of axonemal microtubules, which is absent in primary cilia axonemes (designated as a 9 + 0 structure) but present in motile (9 + 2) cilia^57^. Terminally differentiated ependymal cells and choroid plexus epithelial cells carry motile cilia, however at different stages of development. Ependymal cells differentiate from radial glia, starting at E14.5, but full differentiation occurs only after birth, when they become post-mitotic and form cilia with directional motility^44,55,58^. Ependymal cilia promote CSF flow, e.g., at the medial wall of the foramen of Monro^44,59^. Choroid plexus formation starts earlier (at E11.5) with concomitant formation of transiently motile cilia^60^, which become immotile after birth^61^. Their specific motility role remains to be defined. Primary cilia, in contrast, are immotile and serve as sensory organelles^62^. In the brain, primary cilia mediate sonic hedgehog and Wnt signaling (reviewed in ref. ^63^). Despite these differences between cilia types, multiple structural studies have revealed morphological similarities, and proteome analysis has identified components in common^36,64^. TRIP6 is, apparently, such a component.

During development, radial glia cells give rise to the ependymal cells as well as to less abundant basal cells, some of which are stem cells, that are localized beneath and interspersed within the single-layered epithelial cell layer of the ependyma^28^. In the *trip6*^*−/−*^ mouse, VZ basal cell numbers appear to be reduced. Although speculative at this time, as the number and expression profile of the basal cells remain to be characterized, this observation raises the hypothesis of an earlier developmental origin of the *trip6*^*−/−*^ phenotype. Whether the defects in ependyma and basal cells can be traced back to their development, from primary-cilia carrying radial glia, or to a defect of transcriptional regulation, is a topic that warrants further investigation.

In addition to its ciliary function, TRIP6 also acts as transcription regulator via a nuclear isoform^38,65^ and thus could regulate differentiation programmes. The hypothesis that TRIP6-dependent transcription regulates ependymal differentiation, including ciliogenesis, remains to be tested. In support of this hypothesis, postnatal neuronal progenitor cells, cultured in vitro, suffered from poor proliferation upon TRIP6 knock down^22^. Along similar lines, TRIP6 has been postulated to promote proliferation in cancer (e.g., ref. ^66^; https://www.proteinatlas.org/ENSG00000087077-TRIP6/pathology). However, gross examination of *trip6*^*−/−*^ mouse embryos has not yet revealed any obvious proliferation defects, nor have we detected, with available antibodies, nuclear TRIP6 in brain cells. Nevertheless, in transcription regulation minute protein levels may suffice for large effects. Protein expression below the current detection level might still be required for differentiation of the ependyma. Alternatively, loss of cilia and of their regulatory role could be the primary event that compromises transcriptionally dependent cell maturation. Thus, TRIP6 could exert two functions, in the nucleus and as component of ciliogenesis.

The super resolution microscopy data suggest a molecular function of TRIP6 in the ciliary axoneme. As postulated above, TRIP6 exerts a partially limiting supportive function, either indirectly (via differentiation control) or directly (via a structural role) in cilia formation. A shared feature of motile and primary cilia, mediated by the same protein complexes in both organelles, is the intraflagellar transport (IFT) into and out of the ciliary axoneme^67^ (reviewed in ref. ^68^). Axonemal transport indeed requires organizing factors and scaffold proteins^69^. Thus, disrupting IFT would affect both motile and primary cilia, as we observed upon *trip6* deletion or downregulation. TRIP6 partially co-localized with IFT components (CLUAP1/IFT38 and ARL13B). These data are in agreement with the reported interaction of TRIP6 with CLUAP1 in cytoplasmic complexes of cultured cells^70^, as well as with centrosomal and with cilium-transition-zone proteins^36^ (http://prohits-web.lunenfeld.ca). The increase in “puncta” in the absence of TRIP6 and the altered localization of ciliary components in the resulting structures suggests that ciliation under these conditions is aberrant. The puncta phenotype resembles the ciliogenesis defects caused by deletions of IFT components^37,71^. Finally, in addition to a role in cilia initiation, the punctate localization of TRIP6 along the axoneme, both in vivo and in vitro, is consistent with a function in transport complexes.

Notably, our results demonstrate that TRIP6 function in ciliogenesis requires homodimerization. Dimerization, which doubles the protein interaction sites, most likely enables TRIP6 to interact simultaneously with several cargo proteins—in addition to components of the IFT machinery, CLUAP1/IFT38 and perhaps ARL13B^72^. In support of this conclusion, TRIP6 was localized in the proximity of centriolar satellite and appendage proteins, and within the centrioles themselves, along with proteins of the primary cilium transition zone^36^ (http://prohits-web.lunenfeld.ca). This wide distribution of interactors is a characteristic of an adapter protein/assembly factor. Conversely, our results show that TRIP6 is not required for the replication and translocation of basal bodies in ependymal cells, indicating that the regulatory stimuli inducing cilium formation are not altered.

In conclusion, this study has unveiled a previously unrecognized role for TRIP6 in ciliogenesis, which is critical for brain development. Our findings expand the spectrum of functions of this LIM domain protein beyond the regulation of adhesion, migration, and proliferation. Most importantly, this work has uncovered the essential role of a protein assembly factor in mammalian ciliogenesis.

## Methods

### Generation of trip6 knockout mouse line, colony maintenance and genotyping

Mice carrying *trip6* deleted alleles were generated by ES cell gene targeting as depicted in Supplementary Fig. 2. Briefly, E14.1 mouse embryonic stem (ES) cells were electroporated with the linearized targeting construct, and correct targeting events were identified by Southern-blot hybridization. Selected ES cell clones were subsequently electroporated with the pMC-Cre expression plasmid for deletion of the floxed neo-resistance cassette and generation of clones carrying floxed or deleted alleles of *trip6*. Finally, blastocyst microinjection and transfer into foster mice was employed to generate chimaeras and knockout mouse lines. The resulting *trip6* knockout mouse line was backcrossed to C57BL/6J mice for ten generations.

The *trip6* knockout colony was maintained by breeding heterozygous animals. To avoid genetic drift, routine backcrossing to the parental C57BL/6J strain was employed. The genotype was determined by PCR on DNA extracted from tail tissue using the primers trip6-28F, trip6-29R, and trip6-30R (Supplementary Table 1).

Mouse experiments (including embryo isolation), colony maintenance and breeding were conducted in accordance with Directive 2010/63/EU and the regulations of the Thüringen Landesamt für Verbraucherschutz (Thüringen, Germany) with protocols approved by the respective ethics committee (Tierschutzkomission, Thuringian Animal Welfare Committee) and under the oversight of the FLI Animal Welfare Committee. Animals were provided with standard laboratory chow and tap water ad libitum, and kept at constant temperature (21 °C), with relative air humidity of 55% ± 15 and constant light cycle (12 h-light, 12 h-dark).

### In situ hybridization

A cDNA fragment corresponding to nucleotides 995–1537 of mouse *trip6* cDNA (RefSeq database, accession number NM_011639.3^73^) was generated by PCR and sub-cloned into the pGEM-T Easy Vector (Promega, Madison, WI). Radiolabelled riboprobes were generated by using [^35^S]UTP (Hartmann Analytik, Braunschweig, Germany) as substrate for the in vitro transcription reaction. ISH was carried out according to ref. ^74^. ISH was performed on brain sections of mice aged E14.5, E16, E18, P0, P3 and P6; shown are examples of trip6^+/+^ (hybridization with antisense and sense probe) and trip6^−/−^ (antisense probe).

Briefly, frozen 20 µm thick frontal brain sections were fixed with 4% phosphate-buffered paraformaldehyde solution (pH 7.4) at room temperature (RT) for 60 min and rinsed with PBS (pH 7.4). Tissue sections were treated with 0.4% Triton-PBS for 10 min (RT) and then incubated in 0.1 M triethanolamine (pH 8), containing 0.25% v/v acetic anhydride for 10 min. Following acetylation, sections were rinsed several times with PBS, dehydrated by successive washings with increasing ethanol concentrations, and air-dried.

Radioactive ^35^S-labeled riboprobes were diluted in hybridization buffer (50% formamide, 10% dextran sulfate, 0.6 M NaCl, 10 mM Tris/HCl pH 7.4, 1× Denhardt’s solution, 100 mg/ml sonicated salmon-sperm DNA, 1 mM EDTA-di-Na, and 10 mM dithiothreitol) to a final concentration of 25,000 cpm/µl. After application of the hybridization mix, sections were cover-slipped and incubated in a humid chamber at 58 °C for 16 h. Following hybridization, coverslips were removed and transferred into 2× standard saline citrate (SSC; 0.3 M NaCl, 0.03 M sodium citrate, pH 7.0). The sections were then treated with RNase A (20 mg/ml) and RNase T1 (1 U/ml) at 37 °C for 30 min. Successive washes followed at RT in 1×, 0.5×, and 0.2× SSC for 20 min each and in 0.2× SSC at 60 °C for 1 h.

The tissue was dehydrated and, for microscopic analysis, sections were dipped in KodakNTB nuclear emulsion (Kodak, Rochester, NY) and stored at 4 °C for 32 days. Autoradiograms were developed in Kodak D19 for 5 min, fixed in Rapid Fix (Kodak) for 10 min and analyzed under dark-field illumination. Experiments carried out using the respective sense probe did not produce any hybridization signals.

### Reverse transcription-quantitative PCR (RT-qPCR)

Total RNA was isolated from choroid plexus (4th ventricle) of wild type and *trip6* knockout littermates at P0 using TRIzol Reagent (Ambion, Life Technologies, Cat.# 15596026). Fifty nanogram of total RNA was reverse-transcribed with the iScript cDNA Synthesis Kit (Bio-Rad, Cat.# 1708891) and the cDNA was subjected to qPCR using iQ SYBR Green Supermix (Bio-Rad, Cat.# 1708882) in a thermal cycler (Bio-Rad C1000 Touch Thermal Cycler - CFX384 Real-Time System). The gene-specific primers Trip6-F, Trip6-R, Gapdh-F, and Gapdh-R (Supplementary Table 1) were used. The *trip6* primers are derived from the region of the gene that has been deleted in the *trip6* knockout mice (see Supplementary Fig. 2a). The qPCR results were analysed using the Bio-Rad CFX Manager 3.1 software in the normalized expression (ddCt) mode, with the single threshold Cq determination and baseline subtracted curve fit settings. *Gapdh* (Gene ID: 14433) was used as the reference gene.

### Western blot analysis

Tissues samples were dissected from wild type and *trip6* knockout mice, frozen in liquid nitrogen, homogenized (in a Precellys 24 Homogenizer, Peqlab) in 1× RIPA lysis buffer supplemented with 1 mM phenylmethylsulfonyl fluoride (PMSF) and 1 mM sodium fluoride (NaF), centrifuged (10 min at 14,000 × *g*, 4 °C), fractionated by SDS-PAGE and subjected to western blotting. Lysates from cell cultures were prepared according to ref. ^75^, after RNAi (see below). TRIP6 protein was detected using the anti-TRIP6 A15 antibody (Supplementary Table 3) and an HRP-conjugated secondary antibody (Supplementary Table 4). Unprocessed scans of the uncropped western blots have been included in the Supplementary Information.

### Magnetic resonance imaging (MRI)

To image ventricle size and morphology in early post-natal mice in situ, a high resolution (150 μm isotropic) MRI protocol was applied. Scans were performed on a clinical 3T scanner (Magnetom Trio, Siemens Healthcare), using a dedicated mouse head coil. A Siemens SPACE sequence with a constant flip angle was employed to acquire images with a resolution of 0.2 mm × 0.2 mm × 0.16 mm using the following parameters: echo time TE = 125 ms, repetition time TR = 1900 ms, bandwidth = 130 Hz/px, Turbo Factor TF = 65 and integrated fat saturation. MRI data were processed using the software syngo fastView (Siemens Healthcare). For depiction of multicolored axial images, multiplanar reconstructions from the original 3D MRI data set were assembled.

### Histological processing

Paraffin embedding of mouse brain was performed post fixation in 4% phosphate-buffered paraformaldehyde solution (pH 7.4), at 4 °C, for 48 h. For sagittal sectioning, the brain was cut into two halves along the midline before embedding; for coronal sectioning intact brain was embedded. Haematoxylin & Eosin (H&E) staining was performed according to standard protocol on 5 μm-thick brain sections, prepared from the paraffin-embedded tissue. Images were acquired at an Olympus microscope (VS110 Virtual Slide Scanning System; Olympus; Hamburg, Germany) with a 20× objective.

### Cell culture, ciliogenesis induction, and transfection

The immortalized murine (rat-derived) choroid plexus cell line Z310 (RRID: CVCL_F753) was kindly provided by W. Zheng, Purdue University. Z310 cells were cultured in DMEM high glucose (4.5 g/l) supplemented with 10% FBS and 2 mM glutamine at 37 °C, in 5% CO_2_. The cell line had been characterized by genotyping, morphology, growth assays and expression of the choroidal epithelium-specific marker TTR^34^. Ciliogenesis was induced by starvation [DMEM low glucose (1 g/l), 0% FBS] at 37 °C, in 5% CO_2_, for 24 h.

RNAi experiments were performed using a pool of two ON-TARGETplus siRNAs targeting the human *trip6* open reading frame (Dharmacon). A pool of four non-targeting siRNAs was used as negative control. The siRNA sequences are listed in Supplementary Table 2.

Plasmid transfection was performed with Lipofectamine2000 (Thermo Fisher) according to the manufacturer’s protocol. The pcDNA3.1 constructs encoding the dimerization inhibitory peptide (amino acid 253–265 of human TRIP6, which is conserved between mouse and rat) and the scrambled control peptide tagged with mCherry have been previously described^38^. Z310 cells were transfected with the relevant constructs and, from 48 h of post-transfection, they were starved for 24 h to induce ciliogenesis. At 72 h of post-transfection, the cells were fixed in 3% paraformaldehyde, permeabilized with ice-cold methanol and processed for immunofluorescence labeling^75^.

### Immunofluorescence microscopy

Immunofluorescence labeling was performed on 5 μm-thick paraffin-embedded brain sections from E15.5 mice *(trip6*^*+/−*^ *n* = 4), P0 (*trip6*^*+/−*^ *n* = 2; *trip6*^*−/−*^ *n* = 3) and P4 mice (*trip6*^*+/+*^ *n* = 1; *trip6*^*+/−*^ *n* = 5; *trip6*^*−/−*^ *n* = 6) according to Li et al. 2015 with the following modification: Heat induced antigen retrieval was performed in citrate buffer (10 mM, pH 6.0) in a decloacking chamber (Biocare Medical) by preheating at 95 °C for 10 min followed by 125 °C for 25 min. Antibodies are listed in Supplementary Tables 3 and 4.

Microscopy and image acquisition (Z-stacks) was performed on an Axio Imager microscope (Carl Zeiss Microscopy) equipped with Apotome slider and a 12-bit grayscale cooled CCD AxioCamMRm camera, using a 20× or a 63 × 1.4 NA objective. Representative images were selected from maximum intensity projections (ZEN software, Carl Zeiss Microscopy), brought to a resolution of 300 dpi using Photoshop (Adobe) and the area of interest was cropped. For selected images (Fig. 5) deconvolution was performed (ZEN software, Carl Zeiss Microscopy).

3D reconstruction of the maximum intensity projections was prepared from deconvolved images (Imaris image analysis software, Oxford Instruments). The resulting images are presented as movies (Supplementary Movies 3, 4, and 5). To enable visualization of adhesions in the atrophic area of the trip6^−/−^ ependyma (Supplementary Movie 4), in the β-catenin channel, the intensity histogram was set at 500–11,000 (versus 4400–11,000, for Supplementary Movie 4). Intensity histograms for other channels were set identically, in Supplementary Movies 3 and 4.

### Super-resolution microscopy

Super-resolution imaging was performed on a Leica TCS SP8 X-White Light Laser confocal microscope equipped with an inverted microscope (DMI 8, Leica), a 100× objective (HC PL APO CS2 100 × 1.4 oil), Hybrid detectors and HyVolution-II software. Images were acquired as suggested by the HyVolution module within the LAS microscope software (Leica). Pre-settings in the HyVolution mode were established to achieve the highest possible optical resolution. The super-resolved confocal stacks were deconvolved in Scientific Volume Imaging (SVI)/Huygens deconvolution professional suite software with GPU acceleration.

HyVolution is based on a combination of confocal imaging using sub-Airy pinhole sizes (0.45–0.6 Airy Units, depending on the fluorophores used) with subsequent computational image deconvolution^76,77^. With HyVolution, a lateral resolution of 140 nm can be achieved, which is approximately 1.6 times higher than the resolution achievable with conventional confocal microscopy.

The samples were mounted in ProLong Gold antifade mountant (Thermo Fisher). HyVolution images were acquired using the 488, 546, and 647 nm laser lines of the white-light laser to excite Alexa Fluor 488, Alexa Fluor 546 and Alexa Fluor 647 dyes, respectively. Fluorescence emission was recorded on a hybrid detector at 503–547 nm, 558–604 nm, and 654–719 nm, respectively, employing sequential scan imaging to avoid bleed-through and/or cross-talk between spectral channels. DAPI was excited using a 405 nm laser and detected using a PMT-detector (430–502 nm). Brain sections from P4 mice (*trip6*^*−/+*^ *n* = 2; *trip6*^*−/−*^ *n* = 1) and RNAi-treated Z310 cells (28 cilia from untransfected control group; 20 cilia from *siControl* group; 15 cilia from *siTrip6* group) were imaged.

### Phenotype quantification and statistical analysis

Hydrocephalus incidence, ciliogenesis, and ependymal cell density in vivo, plus ciliogenesis in vitro (in the choroid plexus cell line Z310) were quantified and statistically analysed as described in the sections that follow below. Statistical testing was performed using GraphPad Prism 8 (GraphPad Software, Inc.).

### Hydrocephalus incidence

Hydrocephalus was scored by macroscopic observation of cranial malformations, the incidence was quantified in age-matched cohorts (Supplementary Table 5) and its statistical significance was determined by pairwise chi-squared test (two-tailed) between the genotypes, with Bonferoni correction (*n* = 3 tests, at a *p* < 0.05 level, i.e., *P*_adjusted_ < 0.017). The *trip6−/−* genotype showed a significant influence on the incidence of hydrocephalus versus both controls (*trip6*−/− vs. *trip6*+/−: *p* < 0.0001**;** *trip6*−/− vs. *trip6*+/+: *p* < 0.0001); whereas the incidence in the heterozygous (*trip6*+/−) vs. the wild type (*trip6*+/*+)* showed no significant difference (*p* = 0.2972).

### Ciliogenesis quantification in tissue sections

The analyses were performed using images of H&E stained sagittal brain sections of four control and five *trip6*^*−/−*^ mice (Supplementary Table 6).

For quantification of ependymal cell number and ciliation index, ependymal cells lining the walls of the 4th ventricle were identified by nuclear staining (haematoxylin). Cells were counted for each ventricular wall separately and the length of each wall was measured using the software QuPath (V 0-2-0-m9)^78^.

To determine the distribution of ependymal cells, the cell number was normalized to the length of the segment analysed. The data were tested for normality of distribution and for the difference in variability of ependymal cell numbers between the genotypes (*f*-test, *p* = 0.0026). The log-transformed values were subsequently analysed by two-tailed Welch’s *t*-test (*p* = 0.2713).

The ciliation index was defined as the percentage of ciliated ependymal cells per ventricular section analysed (Supplementary Table 7). The ciliation indices were not normally distributed (by Shapiro–Wilk and B’Agostino-Pearson normality tests). The two-tailed Mann–Whitney test was therefore used to analyse the difference in the median between the genotypes (*p* < 0.0001).

For quantification of the unciliated ependyma segments and of cilia “lawn” height, ciliated, and unciliated areas along the 4th ventricle were analysed. First, continuous regions of interest (ROIs) of 10 µm in length along the ventricular walls were selected. For every second ROI, the height of the cilia lawn was measured at the highest extent of cilia within this ROI. Finally, the ROIs that had a cilia lawn height of 0 μm were counted as unciliated ROIs, normalized to the total number of ROIs in the analysed ventricular section, and presented as unciliated fraction of all measured ROIs per section. Differences between control and *trip6*^*−/−*^ mice were analysed by two-tailed Welch’s *t*-test (*p* < 0.0001).

For quantification of maximum cilia “lawn” height per 10 µm ROI, only ciliated ROIs were considered. The two-tailed Mann–Whitney test was used to analyse the difference in the median between genotypes (*p* < 0.0001).

### Ciliogenesis quantification in Z310 cultured cells

Microscopy and image acquisition were performed as described above using a 63 × 1.4 NA objective. The cilia were imaged as *Z*-stacks encompassing the *Z*-positions from the basal body to the distal tip of the axoneme.

Image based quantification of number of Z310 cells carrying cilia was performed on Maximum Intensity Projections (MIP) for each image, using the ZEN software (Carl Zeiss Microscopy). The “Grid” and “Events” functions from the ZEN (desk) were used to facilitate the counting of the total number of nuclei and the number of cilia in each field. A cilium was counted only when the basal body (visualized by anti-γ-tubulin labeling) and the distal tip of the axoneme (ARL13B) were visible. The cell objects that were not entirely located within the field of view were only counted if carrying a cilium.

In vitro ciliogenesis was quantified in three replicate experiments (Supplementary Table 8). Cilia length was measured using the “spline curve” measurement tool of the ZEN software.

The statistical significance of the difference in the number of cells with primary cilium and in the effect on cilia morphology of s*iControl* vs. *siTrip6* (*p* < 0.0001) and of Control Peptide vs. Blocking Peptide (*p* < 0.0001) treatments was tested by Fisher’s exact test. Cilia length datasets were tested for normality and subsequently analysed by the two tail Student’s *t*-test, for differences in cilia length of *siControl vs. siTrip6* and of Control Peptide vs. Blocking Peptide (*p* < 0.0001) treatments.

### Statistics and reproducibility

Micrographs depict representative examples from experiments that analyzed age-matched cohorts of minimum three animals and from in vitro experiments that had been independently repeated at least three times. For detailed information on replicates, sampling and statistical testing, please refer to the relevant Methods section and Supplementary Tables. The exact *p* values are shown in the figure panels.

### Reporting summary

Further information on research design is available in the Nature Research Reporting Summary linked to this article.

## Supplementary information

Supplementary Information

Description of Additional Supplementary Files

Supplementary Movie 1

Supplementary Movie 2

Supplementary Movie 3

Supplementary Movie 4

Supplementary Movie 5

Reporting Summary

## Data Availability

Source data accompanied this Article. Mouse *trip6* cDNA is available at RefSeq (NM_011639.3). The remaining data are available within the Article and Supplementary Information. Source data are provided with this paper.

## Source data

Source Data

## References

[1] Stepniak E, Radice GL, Vasioukhin V (2009). Adhesive and signaling functions of cadherins and catenins in vertebrate development. Cold Spring Harb. Perspect. Biol..

[2] Kadowaki M (2007). N-cadherin mediates cortical organization in the mouse brain. Dev. Biol..

[3] Stocker AM, Chenn A (2015). The role of adherens junctions in the developing neocortex. Cell Adhes. Migr..

[4] Thumkeo D (2011). Deficiency of mDia, an actin nucleator, disrupts integrity of neuroepithelium and causes periventricular dysplasia. PLoS ONE.

[5] Long KR, Huttner WB (2019). How the extracellular matrix shapes neural development. Open Biol..

[6] Siddiqui MQ, Badmalia MD, Patel TR (2021). Bioinformatic Analysis of structure and function of LIM domains of zyxin family proteins. Int. J. Mol. Sci..

[7] Beckerle MC (1986). Identification of a new protein localized at sites of cell-substrate adhesion. J. Cell Biol..

[8] Gur’ianova OA, Sablina AA, Chumakov PM, Frolova EI (2005). Down-regulation of TRIP6 expression induces actin cytoskeleton rearrangements in human carcinoma cell lines. Mol. Biol..

[9] Bai CY, Ohsugi M, Abe Y, Yamamoto T (2007). ZRP-1 controls Rho GTPase-mediated actin reorganization by localizing at cell-matrix and cell-cell adhesions. J. Cell Sci..

[10] Lin VT, Lin FT (2011). TRIP6: an adaptor protein that regulates cell motility, antiapoptotic signaling and transcriptional activity. Cell. Signal..

[11] Hirata H, Tatsumi H, Sokabe M (2008). Zyxin emerges as a key player in the mechanotransduction at cell adhesive structures. Commun. Integr. Biol..

[12] Smith MA, Hoffman LM, Beckerle MC (2014). LIM proteins in actin cytoskeleton mechanoresponse. Trends Cell Biol..

[13] Dutta S (2018). TRIP6 inhibits Hippo signaling in response to tension at adherens junctions. EMBO Rep..

[14] Zheng Q, Zhao Y (2007). The diverse biofunctions of LIM domain proteins: determined by subcellular localization and protein–protein interaction. Biol. Cell.

[15] James V (2010). LIM-domain proteins, LIMD1, Ajuba, and WTIP are required for microRNA-mediated gene silencing. Proc. Natl Acad. Sci. USA.

[16] Hoffman LM (2003). Targeted disruption of the murine zyxin gene. Mol. Cell Biol..

[17] Vervenne HB (2009). Targeted disruption of the mouse Lipoma Preferred Partner gene. Biochem. Biophys. Res. Commun..

[18] Pratt SJ (2005). The LIM protein Ajuba influences p130Cas localization and Rac1 activity during cell migration. J. Cell Biol..

[19] Feng Y (2007). The LIM protein, Limd1, regulates AP-1 activation through an interaction with Traf6 to influence osteoclast development. J. Biol. Chem..

[20] Moik DV, Janbandhu VC, Fässler R (2011). Loss of migfilin expression has no overt consequences on murine development and homeostasis. J. Cell Sci..

[21] Gray PA (2004). Mouse brain organization revealed through direct genome-scale TF expression analysis. Science.

[22] Lai YJ (2014). TRIP6 regulates neural stem cell maintenance in the postnatal mammalian subventricular zone. Dev. Dyn..

[23] Lv K (2015). Trip6 promotes dendritic morphogenesis through dephosphorylated GRIP1-dependent myosin VI and F-actin organization. J. Neurosci..

[24] Zywitza V, Misios A, Bunatyan L, Willnow TE, Rajewsky N (2018). Single-cell transcriptomics characterizes cell types in the subventricular zone and uncovers molecular defects impairing adult neurogenesis. Cell Rep..

[25] Kousi M, Katsanis N (2016). The genetic basis of hydrocephalus. Annu. Rev. Neurosci..

[26] Wagner C (2003). Cellular mechanisms involved in the stenosis and obliteration of the cerebral aqueduct of hyh mutant mice developing congenital hydrocephalus. J. Neuropathol. Exp. Neurol..

[27] Páez P (2007). Patterned neuropathologic events occurring in hyh congenital hydrocephalic mutant mice. J. Neuropathol. Exp. Neurol..

[28] Jiménez AJ, Domínguez-Pinos MD, Guerra MM, Fernández-Llebrez P, Pérez-Fígares JM (2014). Structure and function of the ependymal barrier and diseases associated with ependyma disruption. Tissue Barriers.

[29] Steiner J (2007). Evidence for a wide extra-astrocytic distribution of S100B in human brain. BMC Neurosci..

[30] Piperno G, Fuller MT (1985). Monoclonal antibodies specific for an acetylated form of alpha-tubulin recognize the antigen in cilia and flagella from a variety of organisms. J. Cell Biol..

[31] Antoniades I, Stylianou P, Skourides PA (2014). Making the connection: ciliary adhesion complexes anchor basal bodies to the actin cytoskeleton. Dev. Cell.

[32] Caspary T, Larkins CE, Anderson KV (2007). The graded response to sonic hedgehog depends on cilia architecture. Dev. Cell.

[33] Gigante ED, Taylor MR, Ivanova AA, Kahn RA, Caspary T (2020). ARL13B regulates Sonic hedgehog signaling from outside primary cilia. eLife.

[34] Zheng W, Zhao Q (2002). Establishment and characterization of an immortalized Z310 choroidal epithelial cell line from murine choroid plexus. Brain Res..

[35] Yang TT (2018). Super-resolution architecture of mammalian centriole distal appendages reveals distinct blade and matrix functional components. Nat. Commun..

[36] Gupta GD (2015). A dynamic protein interaction landscape of the human centrosome-cilium interface. Cell.

[37] Nakamura K (2020). Anterograde transport machinery is required for intraciliary retrograde protein trafficking. J. Biol. Chem..

[38] Diefenbacher ME (2014). The LIM domain protein nTRIP6 recruits the Mediator complex to AP-1-regulated promoters. PLoS ONE.

[39] Botilde Y (2013). Cluap1 localizes preferentially to the base and tip of cilia and is required for ciliogenesis in the mouse embryo. Dev. Biol..

[40] Youn YH, Han Y-G (2018). Primary cilia in brain development and diseases. Am. J. Pathol..

[41] Chu C-W, Ossipova O, Ioannou A, Sokol SY (2016). Prickle3 synergizes with Wtip to regulate basal body organization and cilia growth. Sci. Rep..

[42] Kohli P (2017). The ciliary membrane-associated proteome reveals actin-binding proteins as key components of cilia. EMBO Rep..

[43] Drummond ML (2018). Actin polymerization controls cilia-mediated signaling. J. Cell Biol..

[44] Abdelhamed Z (2018). A mutation in Ccdc39 causes neonatal hydrocephalus with abnormal motile cilia development in mice. Development.

[45] Larkins CE, Aviles GD, East MP, Kahn RA, Caspary T (2011). Arl13b regulates ciliogenesis and the dynamic localization of shh signaling proteins. Mol. Biol. Cell.

[46] Copeland J (2020). Actin-based regulation of ciliogenesis—the long and the short of it. Semin Cell Dev. Biol..

[47] Mirvis M, Stearns T, Nelson WJ (2018). Cilium structure, assembly, and disassembly regulated by the cytoskeleton. Biochem. J..

[48] Uddin B (2019). The human phosphatase CDC14A modulates primary cilium length by regulating centrosomal actin nucleation. EMBO Rep..

[49] Willems PJ, Brouwer OF, Dijkstra I, Wilmink J (1987). X-linked hydrocephalus. Am. J. Med. Genet..

[50] Rosenthal A, Jouet M, Kenwrick S (1992). Aberrant splicing of neural cell adhesion molecule L1 mRNA in a family with X-linked hydrocephalus. Nat. Genet..

[51] Tissir F (2010). Lack of cadherins Celsr2 and Celsr3 impairs ependymal ciliogenesis, leading to fatal hydrocephalus. Nat. Neurosci..

[52] Yamamoto H (2013). Genetic deletion of afadin causes hydrocephalus by destruction of adherens junctions in radial glial and ependymal cells in the midbrain. PLoS ONE.

[53] Guerra MM (2015). Cell junction pathology of neural stem cells is associated with ventricular zone disruption, hydrocephalus, and abnormal neurogenesis. J. Neuropathol. Exp. Neurol..

[54] Kim J (2015). Actin remodelling factors control ciliogenesis by regulating YAP/TAZ activity and vesicle trafficking. Nat. Commun..

[55] Narita K, Takeda S (2015). Cilia in the choroid plexus: their roles in hydrocephalus and beyond. Front. Cell. Neurosci..

[56] Malicki JJ, Johnson CA (2017). The cilium: cellular antenna and central processing unit. Trends Cell Biol..

[57] Davenport JR, Yoder BK (2005). An incredible decade for the primary cilium: a look at a once-forgotten organelle. Am. J. Physiol..

[58] Spassky N (2005). Adult ependymal cells are postmitotic and are derived from radial glial cells during embryogenesis. J. Neurosci..

[59] Olstad EW (2019). Ciliary beating compartmentalizes cerebrospinal fluid flow in the brain and regulates ventricular development. Curr. Biol..

[60] Nonami Y, Narita K, Nakamura H, Inoue T, Takeda S (2013). Developmental changes in ciliary motility on choroid plexus epithelial cells during the perinatal period. Cytoskeleton.

[61] Inoue T, Narita K, Nonami Y, Nakamura H, Takeda S (2015). Observation of the ciliary movement of choroid plexus epithelial cells ex vivo. J. Vis. Exp..

[62] Goetz SC, Anderson KV (2010). The primary cilium: a signalling centre during vertebrate development. Nat. Rev. Genet..

[63] Park SM, Jang HJ, Lee JH (2019). Roles of primary cilia in the developing brain. Front. Cell. Neurosci..

[64] Narita K (2012). Proteomic analysis of multiple primary cilia reveals a novel mode of ciliary development in mammals. Biol. Open.

[65] Kemler D, Dahley O, Roßwag S, Litfin M, Kassel O (2016). The LIM domain protein nTRIP6 acts as a co-repressor for the transcription factor MEF2C in myoblasts. Sci. Rep..

[66] Miao X (2016). Overexpression of TRIP6 promotes tumor proliferation and reverses cell adhesion-mediated drug resistance (CAM-DR) via regulating nuclear p27(Kip1) expression in non-Hodgkin’s lymphoma. Tumour Biol..

[67] Madhivanan K, Aguilar RC (2014). Ciliopathies: the trafficking connection. Traffic.

[68] Reiter JF, Blacque OE, Leroux MR (2012). The base of the cilium: roles for transition fibres and the transition zone in ciliary formation, maintenance and compartmentalization. EMBO Rep..

[69] Horani A (2018). Establishment of the early cilia preassembly protein complex during motile ciliogenesis. Proc. Natl Acad. Sci. USA.

[70] Beyer T (2018). CRISPR/Cas9-mediated genomic editing of Cluap1/IFT38 reveals a new role in actin arrangement. Mol. Cell Proteom..

[71] Hirano T, Katoh Y, Nakayama K (2017). Intraflagellar transport-A complex mediates ciliary entry as well as retrograde trafficking of ciliary G protein-coupled receptors. Mol. Biol. Cell.

[72] Nozaki S (2017). Regulation of ciliary retrograde protein trafficking by the Joubert syndrome proteins ARL13B and INPP5E. J. Cell Sci..

[73] O’Leary NA (2016). Reference sequence (RefSeq) database at NCBI: current status, taxonomic expansion, and functional annotation. Nucleic Acids Res..

[74] Heuer H, Schäfer MK, O’Donnell D, Walker P, Bauer K (2000). Expression of thyrotropin-releasing hormone receptor 2 (TRH-R2) in the central nervous system of rats. J. Comp. Neurol..

[75] Li H (2016). Impaired planar germ cell division in the testis, caused by dissociation of RHAMM from the spindle, results in hypofertility and seminoma. Cancer Res..

[76] Schrader M, Hell SW, van der Voort HTM (1996). Potential of confocal microscopes to resolve in the 50–100 nm range. Appl Phys. Lett..

[77] Lam F, Cladière D, Guillaume C, Wassmann K, Bolte S (2017). Super-resolution for everybody: an image processing workflow to obtain high-resolution images with a standard confocal microscope. Methods.

[78] Bankhead P (2017). QuPath: Open source software for digital pathology image analysis. Sci. Rep..

