# Supplementary material for: TRIP6 functions in brain ciliogenesis
Source: Nat Commun. 2021 Oct 7;12:5887. doi: 10.1038/s41467-021-26057-6 (PMC8497538; doi:10.1038/s41467-021-26057-6)
Supplement: Supplementary file 1 — Supplementary Information [file 41467_2021_26057_MOESM1_ESM.pdf]

TRIP6 functions in brain ciliogenesis

Shalmali Shukla, Ronny Haenold, Pavel Urbánek, Lucien Frappart, Shamci Monajembashi, Paulius Grigaravicius, Sigrun Nagel, Woo Kee Min, Alicia Tapias, Olivier Kassel, Heike Heuer, Zhao-Qi Wang, Aspasia Ploubidou, Peter Herrlich

---

Supplementary Information

The Supplementary Information comprises 6 Figures and 8 Tables (presented in this document), plus 5 Movies that can be accessed via the journal webpage.

Supplementary Figure 1:
ISH controls; TRIP6 expression in embryonic and postnatal brain.

Supplementary Figure 2:
Generation of the *trip6-/-* mouse line.

Supplementary Figure 3:
Defective differentiation of ependyma and choroid plexus in *trip6*$^{-/-}$mice.

Supplementary Figure 4:
Reduced S100β expression and ciliation in the ependyma of *trip6*$^{-/-}$ mice.

Supplementary Figure 5:
TRIP6 localisation in adhesion complexes and cilia, in choroid plexus-derived Z310 epithelial cells.
Downregulation of trip6 by siRNA, in the choroid plexus-derived cell line Z310, does not impair formation of focal adhesions or adherens junctions.

Supplementary Figure 6:
Downregulation of *trip6* by siRNA results in aberrant ciliogenesis, *in vitro.*

Supplementary Table 1:
Primer sequences.

Supplementary Table 2:
siRNA description.

Supplementary Table 3:
Primary antibodies information.

Supplementary Table 4:
Secondary antibodies information.

Supplementary Table 5:
Quantification of hydrocephalus incidence.

Supplementary Table 6:
Cilia quantification in tissue sections.

Supplementary Table 7:
Cilia quantification parameters.

Supplementary Table 8:
Cilia quantification in Z310 cultured cells.

Supplementary Movie 1:
MRI of trip6+/- mouse brain

Supplementary Movie 2:
MRI of trip6-/- mouse brain

Supplementary Movie 3:
Animated 3D reconstruction of the ependyma, lining the LV, in a control (*trip6*$^{+/-}$) mouse.

Supplementary Movie 4:
Animated 3D reconstruction of the ependyma, lining the LV, in a *trip6*$^{-/-}$ mouse.

Supplementary Movie 5:
Animated 3D reconstruction of the ependyma, lining the LV, in a *trip6*$^{-/-}$ mouse.

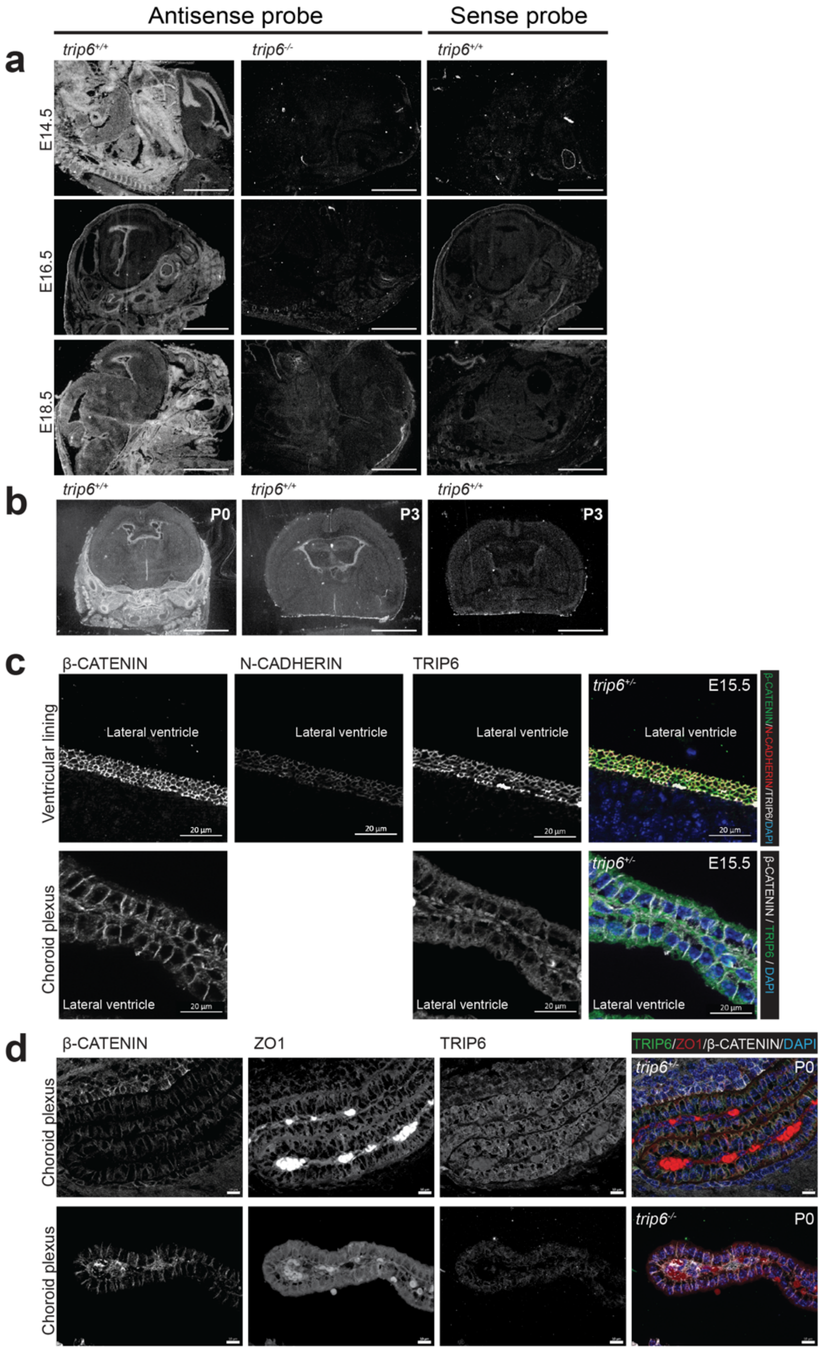

Supplementary Figure 1:

ISH controls; TRIP6 expression in embryonic and postnatal brain.

(a) *trip6* mRNA expression determined by ISH. Embryonic sagittal sections, through the entire skull of *trip6*$^{+/+}$ and *trip6*$^{-/-}$ mice, were hybridized with *trip6* sense and antisense probes. Hybridization with the anti-sense probe on *trip6*$^{-/-}$ sections serves as proof of *trip6* deletion, whilst hybridization with the sense probe on *trip6*$^{+/+}$ sections as negative control of the ISH.

(b) Postnatal coronal sections, ISH as in A. The specimens depicted are from littermates of the animals used in the experiment presented in Figure 1, thus further confirming the ISH control data.

(c) Immunofluorescence microscopy of VZ cells and choroid plexus epithelium from *trip6*$^{+/-}$ E15.5 brain demonstrate co-staining of cellular membranes with β-catenin, N-cadherin and TRIP6. The oblique sections through the VZ enable visualisation of several adjacent VZ cells.

(d) Immunofluorescence microscopy of β-catenin, ZO1 and TRIP6 co-labelling in choroid plexus sections of *trip6*$^{+/-}$ (repetition of the data shown in Figure 1C), now in comparison to choroid plexus sections of *trip6*$^{-/-}$ mice (all P0). Absence of TRIP6 did not change the cell adhesions (labelled by β-catenin) and tight junctions (labelled by ZO1). (Ignore the non-specific staining by the anti-ZO1 antibody in the choroid plexus vasculature).

Scale bars: 1 mm (**a, b**), 20µm (**c**), 10µm (**d**).

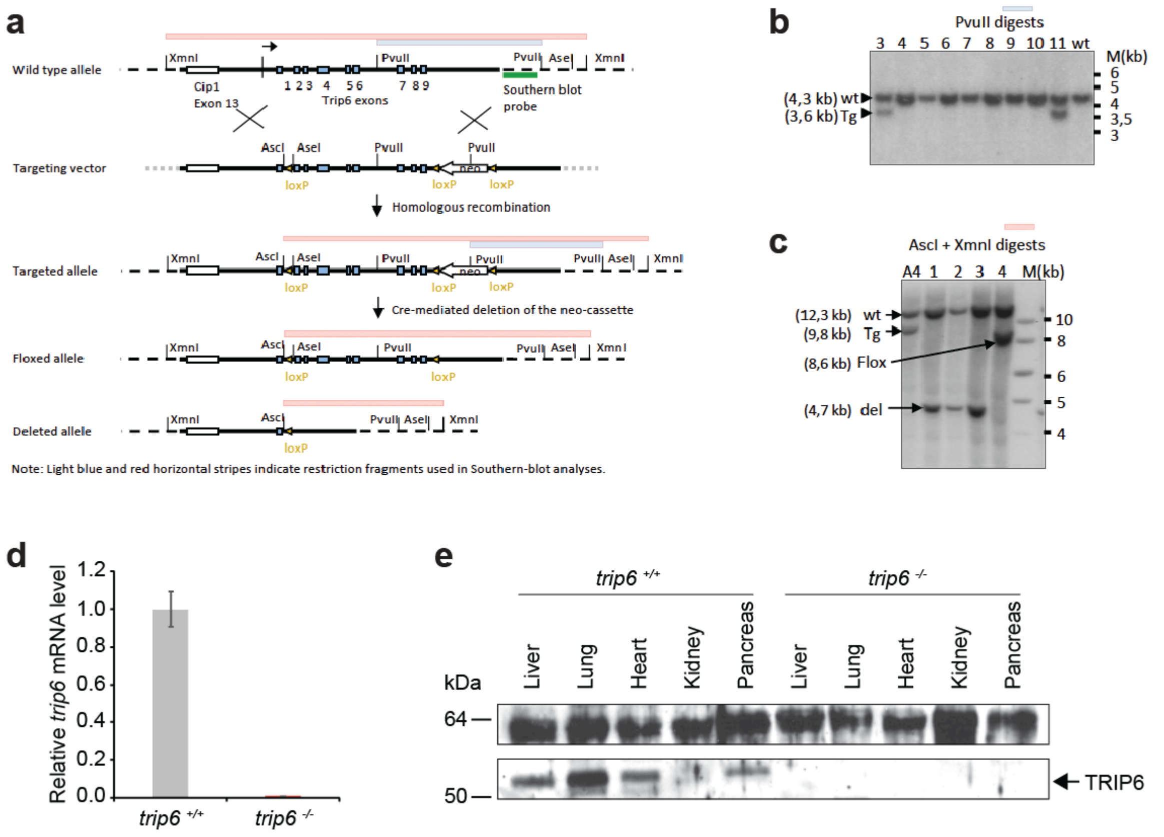

Supplementary Figure 2:
Generation of the *trip6-/-* mouse line.
(**a**) *trip6* gene targeting strategy. Southern blot analysis of *trip6* targeted (**b**), *trip6* deleted and *trip6* floxed (**c**) clones. (**d**) Deletion of *trip6* was confirmed at mRNA level, in choroid plexus, via RT-qPCR (n= 3; data are presented as mean values ±SEM) (**d**) and at protein level, in several organs, via immunoblotting (**e**). [In addition to TRIP6 binding, the primary antibody cross-reacts with an approximately 64 kDa protein (**e**) that has been used as internal loading control]. Source data are provided as a Source Data file.

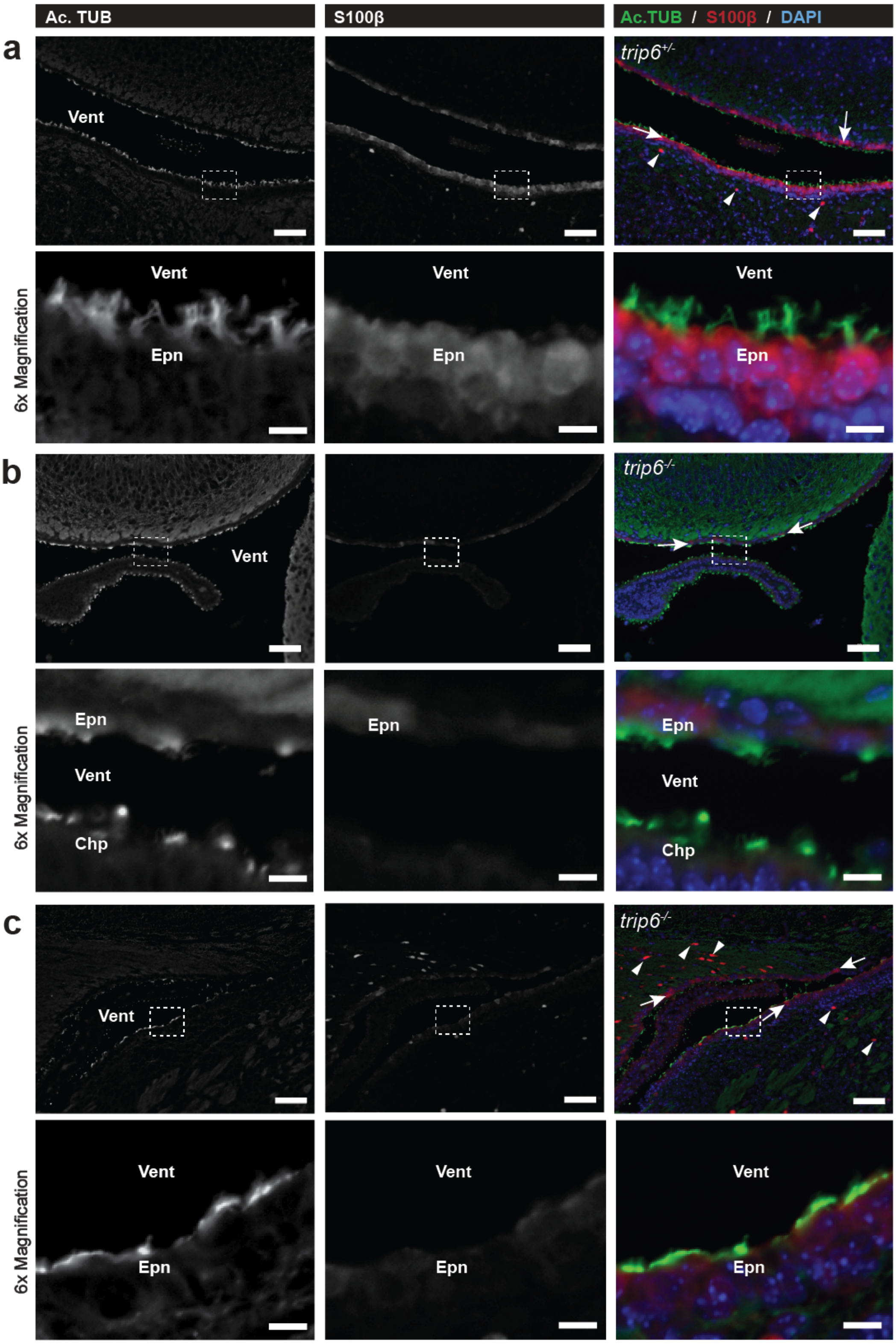

Supplementary Figure 3:

Defective differentiation of ependyma and choroid plexus in *trip6*$^{-/-}$mice.

Selected panels (d, e, f) from Figure 4 are shown here again (as **a, b, c,** correspondingly) accompanied with higher magnification images of the framed areas, for improved visualization of the cilia (Ac.TUB) and ependymal cell differentiation (S100β) markers. (Chp: choroid plexus; Epn: ependyma; Vent: ventricular lumen). Scale bars: 50μm (low magn. panels of **a, b, c**), 10μm (high magn. panels of **a, b, c**).

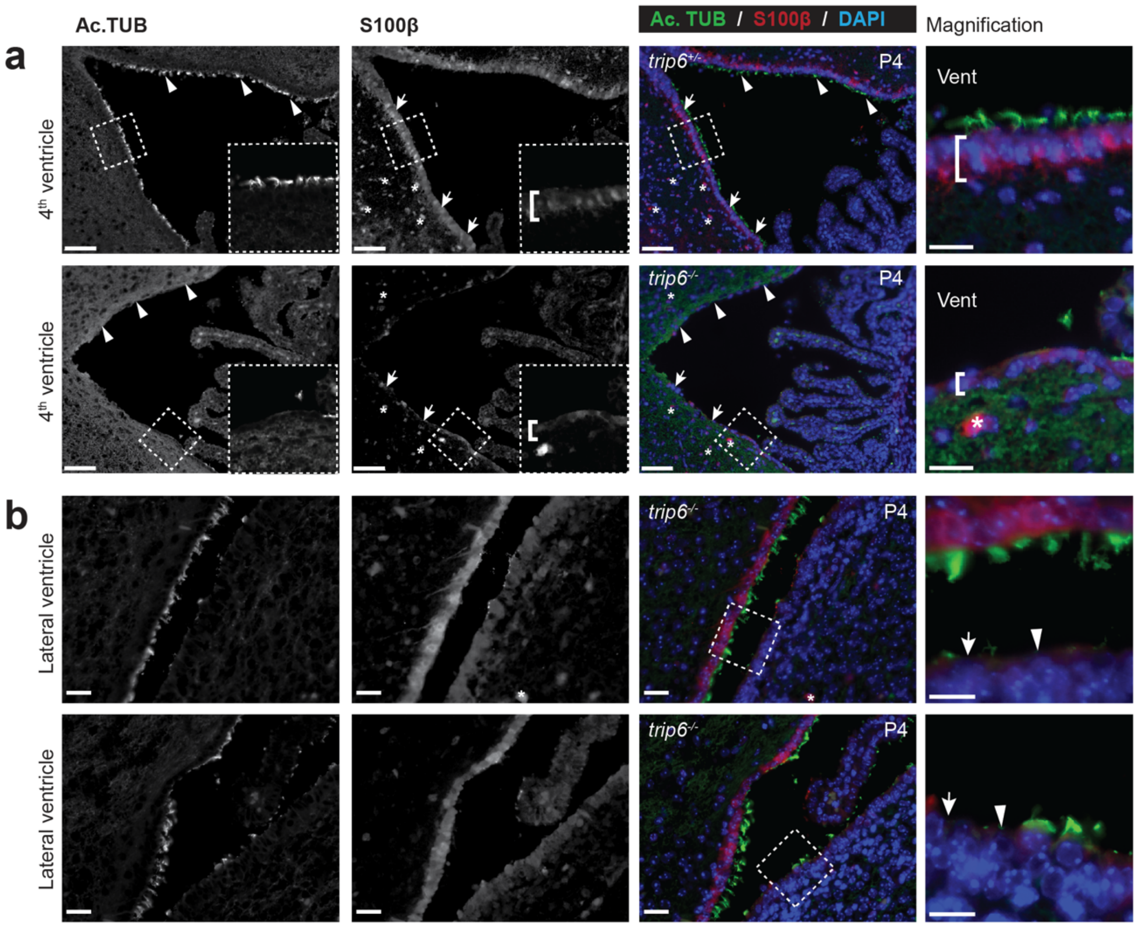

Supplementary Figure 4:
Reduced S100β expression and ciliation in the ependyma of *trip6*$^{-/-}$ mice.
Sections through the 4th ventricle (**a**) or the LV (**b**) of *trip6*$^{+/-}$ and *trip6*$^{-/-}$ mouse brain (all at P4) were co-labelled with anti-S100β and anti-acetylated α-tubulin (Ac.TUB) antibodies to visualise the ependymal cells (arrows indicating S100β expressing cells in *trip6*$^{+/-}$ or their absence in *trip6-/-*) and their cilia (arrowheads indicate cilia in *trip6*$^{+/-}$ or their absence in *trip6*$^{-/-}$). Ependymal layer thickness is compared (brackets). * indicates non-ependymal cell labelled with anti-S100β. The *trip6*$^{-/-}$ ependymal layer exhibits reduction in S100β expression, consistent with poor differentiation presenting with an endothelial aspect (i.e. flattened cell morphology; brackets in **A** indicate the thickness of the epithelium), concomitantly with aberrant/no ciliation. In (**a**), * indicate S100β expressing cells outside the ependymal layer (likely glia cells), VENT = ventricular lumen. Scale bars: 10µm (**a**, low magn.), 50µm (**b**, low magn.), 2µm (**a**, high magn.), 10µm (**b**, high magn.)

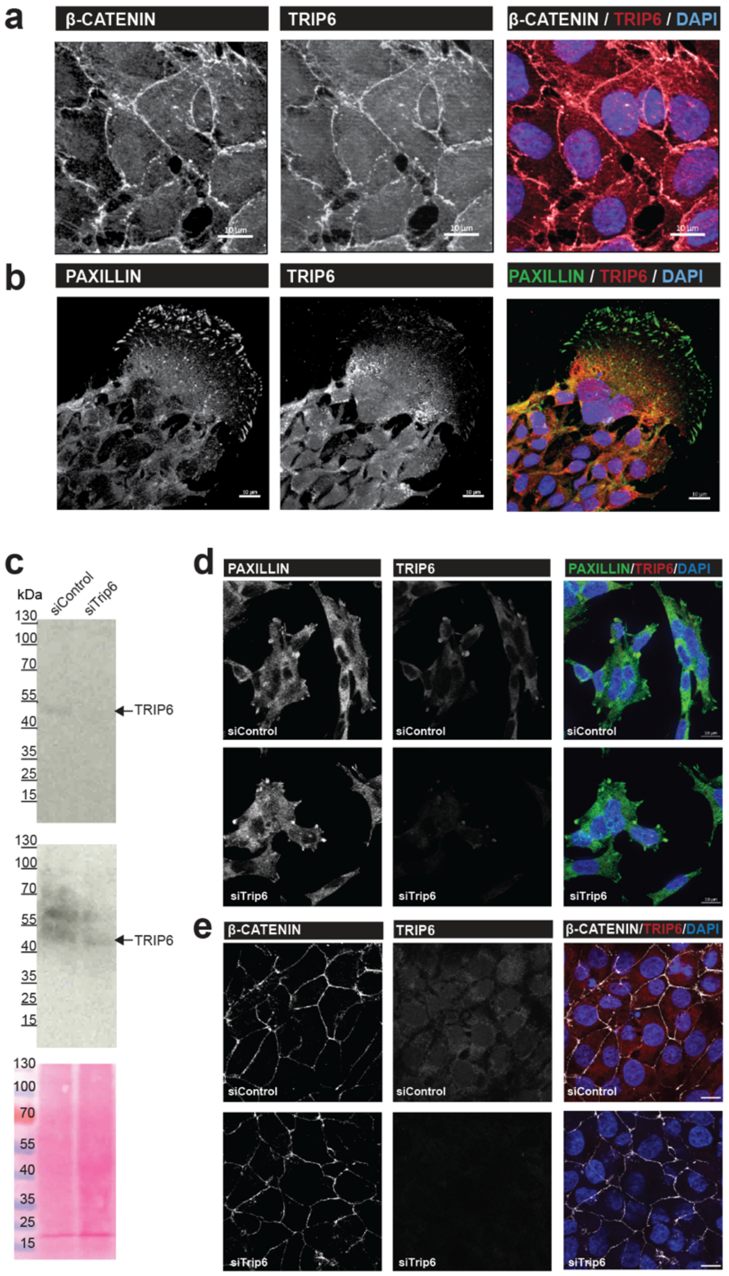

Supplementary Figure 5:

TRIP6 localisation in adhesion complexes and cilia, in choroid plexus-derived Z310 epithelial cells (a,b).

Immunofluorescence microscopy demonstrates the co-localisation of TRIP6 with β-catenin at adherens junctions (**a**) and with paxillin at focal adhesions (**b**), in cultured Z310 cells. Scale bars: 10µm.

Downregulation of trip6 by siRNA, in the choroid plexus-derived cell line Z310, does not impair formation of focal adhesions or adherens junctions (c,d,e).

Z310 cells, transfected with the indicated siRNAs and serum-starved to induce primary cilium formation (see Methods for detailed description), were analysed by western blotting (**c**) or by immunofluorescence microscopy (**d, e**). TRIP6 protein downregulation, of ca. 50%, was determined by western blotting (**c**). Two exposures of different duration, of the same w. blot, are shown, as well as the Ponceau-stained membrane that indicates protein loading. The downregulation of *trip6* (*siTrip6*) impaired ciliogenesis in Z310 cells (shown in Figure 8) but had no effect on their focal adhesion (visualised by anti-paxillin labelling, **d**) or adherens junctions formation (visualised by anti-β-catenin labelling, **e**), as demonstrated by comparison to *siControl* treated cells. (The cultures depicted in **d** and **e** have been grown to different cell densities, as formation of adherens junctions requires relatively high cell density, while focal adhesions are optimally visualised at lower cell density). Source data are provided as a Source Data file. Scale bars: 10µm.

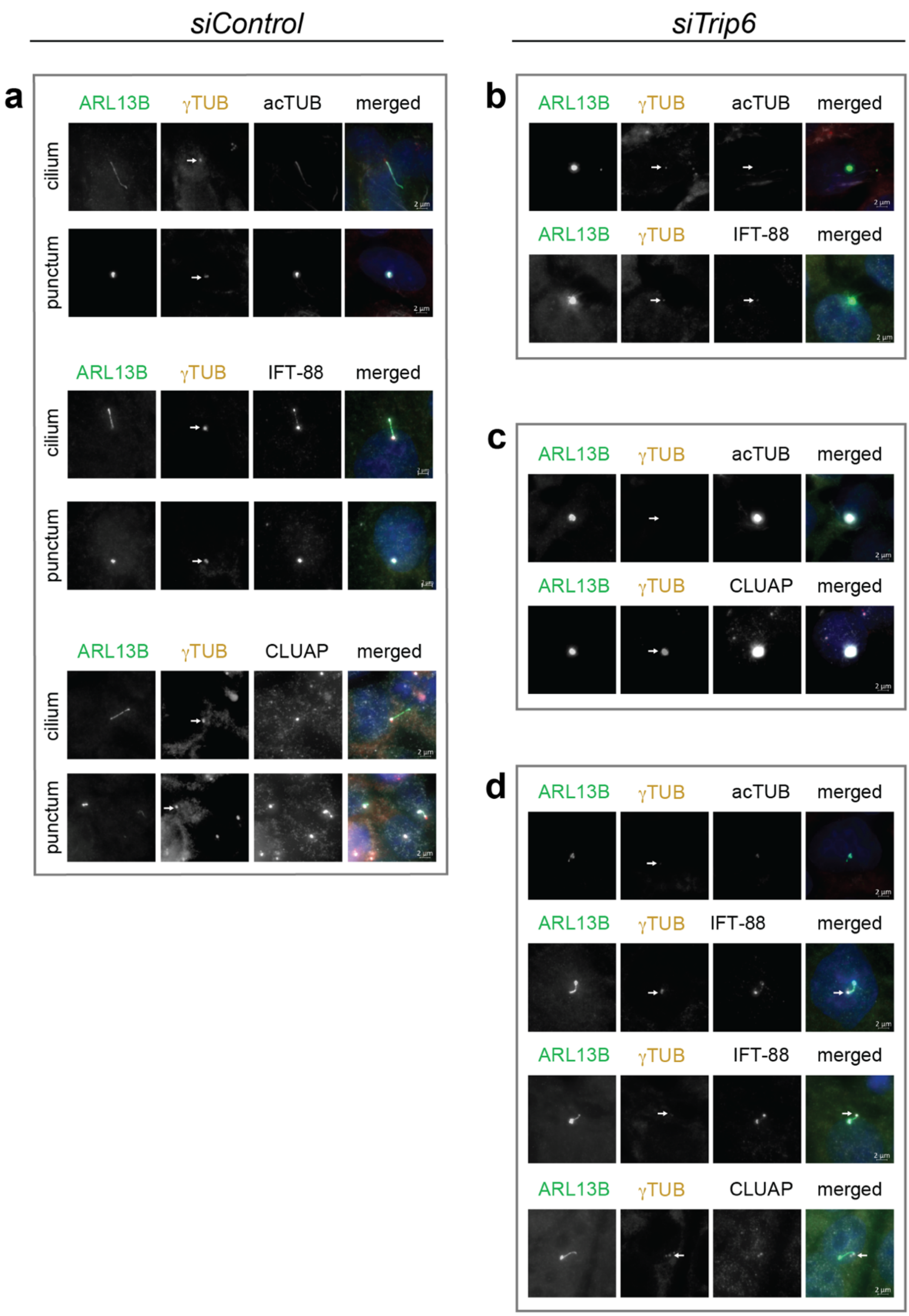

Supplementary Figure 6:

Downregulation of *trip6* by siRNA results in aberrant ciliogenesis, *in vitro.*

Immunofluorescence microscopy analysis of cilia and "puncta" from siControl **(a)** and siTrip6 **(b, c, d)** treated Z310 cells, labelled with the antibodies indicated (arrows point to the basal body/centrosome). The micrographs depict selected examples of incomplete or abortive ciliation (puncta). Exposure times were adjusted individually for each cilium, for optimal visualisation of the phenotypes; intensities are therefore not indicative of a relative quantitative readout. Scale bars: 2μm.

Supplementary Table 1	
**Primer name**	**Primer sequence**
trip6-28F	5´-TCA CCT TTT CTC CCT TGC CTG CCT-3´
trip6-29R	5´-GGT ACC CCC GGA GGC TGA TAA CAG-3´
trip6-30R	5´-GCT TAT CGA TAC CGT CGA CCT CGA-3´
Trip6-F	5´-GCT TTG TGT GTT CTA CCT GTC G-3´
Trip6-R	5´-GAT GGG TTC AGA GCA TGT GGA A-3'
Gapdh-F	5´-CAA AAT GGT GAA GGT CGG TGT G-3´
Gapdh-R	5´-GTT GAA TTT GCC GTG AGT GGA G-3´

Supplementary Table 1:
Primer sequences. Please see Methods (sections *Generation of trip6 knockout mouse line, colony maintenance and genotyping; Reverse transcription - quantitative PCR (RT-qPCR)*).

<table>
<tr><th colspan="4">Supplementary Table 2</th></tr>
<tr><th>Target</th><th>Sense sequence of siRNA duplexes</th><th>Dharmacon catalogue Nr.</th><th>NCBI BLAST alignment to R. norvegicus (RefSeq NM_001127570.1)</th></tr>
<tr><td>Trip6</td><td>GAUCGAAGUUUUCACAUUG</td><td>J-012605-05</td><td>1 GATCGAAGTTTTCACATTG 19
||| ||||||| |||||||
1425 GATAGAAGTTTCCACATTG 1443</td></tr>
<tr><td>Trip6</td><td>GGAGGAGACUGUGAGAAUU</td><td>J-012605-08</td><td>1 GGAGGAGACTGTGAGAATT 19
||||||||| |||||||||
1397 GGAGGAGACAGTGAGAATT 1415</td></tr>
<tr><td>Non-Targeting</td><td>UAAGGCUAUGAAGAGAUAC</td><td>D-001210-02</td><td></td></tr>
<tr><td>Non-Targeting</td><td>AUGUAUUGGCCUGUAUUAG</td><td>D-001210-03</td><td></td></tr>
<tr><td>Non-Targeting</td><td>AUGAACGUGAAUUGCUCAA</td><td>D-001210-04</td><td></td></tr>
<tr><td>Non-Targeting</td><td>UGGUUUACAUGUCGACUAA</td><td>D-001210-05</td><td></td></tr>
</table>

Supplementary Table 2:
siRNA description. Please see Methods (section *Cell culture, ciliogenesis induction, transfection*).

Supplementary Table 3			
**Primary antibody**	**Raised in**	**Supplier (Cat#)**	**Dilution**
Anti-Acetylated α-Tubulin	mouse	Sigma (T6793)	1:100
Anti-ARL13B	mouse	Proteintech (17/11-1-AP)	1:100
Anti-ARL13B	rat	BiCell Scientific Inc (90413)	1:100
Anti-β-Catenin	rabbit	Sigma (C2206)	1:500
Anti-β-Catenin (12F7)	mouse	Santa Cruz Biotechnology (sc-59737)	1:100
Anti-CLUAP1	rabbit	Proteintech (17470-1-AP)	1:100
Anti-γ-Tubulin (GTU88)	mouse	Sigma (T6557)	1:200
Anti-N-cadherin (H-63)	rabbit	Santa Cruz Biotechnology (sc-7939)	1:100
Anti-IFT-88	rabbit	Proteintech (13967-I-AP)	1:2,000
Anti-Paxillin	mouse	Transduction Lab (P13520)	1:1000
Anti-Pericentrin	rabbit	Biolegend (923701)	1:100
Anti-S100β	rabbit	DAKO (GA504)	1:100
Anti-Trip6 (A15)	goat	Santa Cruz Biotechnology (sc-34976)	1:50
Anti-ZO1	rabbit	Abcam (ab59720)	1:100

Supplementary Table 3:
Primary antibodies information. Please see Methods (sections *Immunofluorescence microscopy; Western blot analysis*).

Supplementary Table 4			
**Secondary antibody**	**Raised in**	**Supplier (Cat#)**	**Dilution**
Anti-mouse IgG (H+L) Highly Cross-Adsorbed, Alexa Fluor 546 conjugated	donkey	Life technologies (A10036)	1:100
Anti-goat IgG (H+L) Cross-Adsorbed, Alexa Fluor 546 conjugated	donkey	Life technologies (A11056)	1:100
Anti-rabbit IgG (H+L) Highly Cross-Adsorbed, Alexa Fluor 546 conjugated	donkey	Life technologies (A10040)	1:100
Anti-mouse IgG (H+L) Highly Cross-Adsorbed, Alexa Fluor 488 conjugated	donkey	Life technologies (A21202)	1:100
Anti-goat IgG (H+L) Cross-Adsorbed, Alexa Fluor 488 conjugated	donkey	Life technologies (A11055)	1:100
Anti-rabbit IgG (H+L) Highly Cross-Adsorbed, Alexa Fluor 488 conjugated	donkey	Life technologies (A21206)	1:100
Anti-rat IgG (H+L) Highly Cross-Adsorbed, Alexa Fluor 488 conjugated	goat	Invitrogen (A-11006)	1:300
Anti-rabbit IgG (H+L) Highly Cross-Adsorbed, Alexa Fluor 647 conjugated	donkey	Life technologies (A31573)	1:100
Anti-mouse IgG (H+L) Highly Cross-Adsorbed, Alexa Fluor 647 conjugated	donkey	Life technologies (A31571)	1:100
Anti-goat IgG (H+L) HRP-conjugated	rabbit	Invitrogen (31402)	1:10000

Supplementary Table 4:
Secondary antibodies information. Please see Methods (sections *Immunofluorescence microscopy; Western blot analysis*).

Supplementary Table 5							
**Genotype**	**Age range** (days)	**Mouse cohort analysed**			**Mice presenting with hydrocephalus**		
		**Total Nr. of mice**	*Nr. of females*	*Nr. of males*	**Total Nr. of mice**	*Nr. of females*	*Nr. of males*
trip6$^{+/+}$	5 - 417	**214**	*114*	*100*	**1**	*1*	*0*
trip6$^{+/-}$	3 - 460	**232**	*120*	*112*	**0**	*0*	*0*
trip6$^{-/-}$	3 - 425	**243**	*127*	*116*	**107**	*62*	*45*

Supplementary Table 5:
Information on mice scored in quantification of hydrocephalus incidence. Please see Methods (section *Hydrocephalus incidence*).

Supplementary Table 6		
**Mouse genotype**	**Mouse age** (post-natal days)	**Nr. of brain sections analysed**
-/-	P21	2
-/-	P17	4
-/-	P15	4
-/-	P14	4
-/-	P13	2
+/+	P21	4
+/+	P17	4
+/-	P15	2
+/-	P14	4

Supplementary Table 6:
Information on tissue sections used for cilia quantification. Please see Methods (section *Ciliogenesis quantification in tissue sections*).

Supplementary Table 7								
**Sampling Parameters**	**Quantification Parameters**							
	**Ependymal cell density**		**Ciliation index**		**Unciliated ependyma**		**Cilia lawn height**	
	control	$trip6^{-/-}$	control	$trip6^{-/-}$	control	$trip6^{-/-}$	control	$trip6^{-/-}$
Nr. of mice	4	5	4	5	4	5	4	5
Nr. of sections	14	16	14	16	14	16	14	16
Sampling unit description	Nr. cells/100µm		Total Nr. of sections		Nr. of un-ciliated (10µm) segments		Cilia lawn height (µm)/10µm segment	
Sampling unit Nr. (n_1)	52	53	14	16	14	16	521	540
Sample size (n_2)	1196 cells	1253 cells	1568 cells	1203 cells	73 ROIs	229 ROIs	448 ROIs	311 ROIs

Supplementary Table 7:
Information on cilia quantification parameters. Please see Methods (section *Ciliogenesis quantification in tissue sections*).

Supplementary Table 8								
	**siControl**				**siTrip6**			
Experiment Replicate Nr.	***Nr. of cells analysed***	***ciliated cells***	***cells with puncta***	***Nr. of cilia analysed for length***	***Nr. of cells analysed***	***ciliated cells***	***cells with puncta***	***Nr. of cilia analysed for length***
1	1039	197	18		709	88	38	
2	1407	267	71	46	1266	67	116	25
3	541	98	18	13	446	35	74	50
total	***2987***	***562***	***107***	***59***	***2421***	***190***	***228***	***75***
	**Control Peptide**				**Blocking Peptide**			
Experiment Replicate Nr.	***Nr. of cells analysed***	***ciliated cells***	***cells with puncta***	***Nr. of cilia analysed for length***	***Nr. of cells analysed***	***ciliated cells***	***cells with puncta***	***Nr. of cilia analysed for length***
1	826	100		51	692	42		46
2	127	40	8	11	123	11	33	10
3	287	30	13		352	26	48	
total	***1240***	***170***	***21***	***62***	***1167***	***59***	***81***	***56***

Supplementary Table 8:
Information on *in vitro* ciliogenesis experiments. Please see Methods (section *Ciliogenesis quantification in Z310 cultured cells*).
