# Supplementary material for: TRIP6 functions in brain ciliogenesis
Source: Nat Commun. 2021 Oct 7;12:5887. doi: 10.1038/s41467-021-26057-6 (PMC8497538; doi:10.1038/s41467-021-26057-6)
Supplement: Supplementary file 2 — Description of Additional Supplementary Files [file 41467_2021_26057_MOESM2_ESM.pdf]

Description of Additional Supplementary Files

File Name: Supplementary Movie 1

Description: **MRI of trip6+/- mouse brain**

Animated maximum intensity projection (MIP) reconstructions through the brain of a control (trip6$^{+/-}$) mouse, at P21, depicting the spatial organization of the normal ventricular system. Selected still frames are presented in Figure 2e.

File Name: Supplementary Movie 2

Description: **MRI of trip6-/- mouse brain**

Animated maximum intensity projection (MIP) reconstructions through the brain of a trip6$^{-/-}$ mouse, at P21, depicting the enormously enlarged LV as well as the unaffected 3rd and 4th ventricles. Selected still frames are presented in Figure 2e.

File Name: Supplementary Movie 3

Description: **Animated 3D reconstruction of the ependyma, lining the LV, in a control (*trip6*$^{+/-}$) mouse.**

Immunofluorescence microscopy was performed as described in Figure 5. Briefly, cilia and adherence junctions were labelled with anti-acetylated α-tubulin (shown pseudo-coloured in green) and anti-β-catenin (shown in white) antibodies, respectively. Nuclei are stained with DAPI (in blue).

Note the regularly spaced cilia clusters along the ependymal cell layer, and the “mesh” of adherence junctions inter-connecting the ependymal cells in the VZ.

The deconvolved image of this area can be seen in Figure 5a.

Scale bar: 10µm.

File Name: Supplementary Movie 4

Description: **Animated 3D reconstruction of the ependyma, lining the LV, in a *trip6*$^{-/-}$ mouse.**

Immunofluorescence microscopy was performed as described in Figure 5. Briefly, cilia and adherence junctions were labelled with anti-acetylated α-tubulin (shown pseudo-coloured in green) and anti-β-catenin (shown in white) antibodies, respectively. Nuclei are stained with DAPI (in blue).

Note the aberrant cilia coverage along the ependymal cell layer, and the “mesh” of adherence junctions inter-connecting the ependymal cells in the VZ.

The deconvolved image of this area can be seen in Figure 5b.

Scale bar: 10µm.

File Name: Supplementary Movie 5

Description: **Animated 3D reconstruction of the ependyma, lining the LV, in a *trip6*$^{-/-}$ mouse.**

Immunofluorescence microscopy was performed as described in Figure 5. Briefly, basal bodies and adherence junctions were labelled with anti-γ-tubulin (shown pseudo-coloured in green) and anti-β-catenin (shown in white) antibodies, respectively. Nuclei are stained with DAPI (in blue).

Note the basal body clusters on each ependymal cell, and the “mesh” of adherence junctions inter-connecting the ependymal cells in the VZ.

The deconvolved image of this area can be seen in Figure 5f.

Scale bar: 5µm.
