# Supplementary material for: TRIP6 functions in brain ciliogenesis
Source: Nat Commun. 2021 Oct 7;12:5887. doi: 10.1038/s41467-021-26057-6 (PMC8497538; doi:10.1038/s41467-021-26057-6)
Supplement: Supplementary file 8 — Reporting Summary [file 41467_2021_26057_MOESM8_ESM.pdf]

nature research

Corresponding author(s): Peter Herrlich, Aspasia Ploubidou

Last updated by author(s): July 24th, 2021

# Reporting Summary

Nature Research wishes to improve the reproducibility of the work that we publish. This form provides structure for consistency and transparency in reporting. For further information on Nature Research policies, see Authors & Referees and the Editorial Policy Checklist .

## Statistics

For all statistical analyses, confirm that the following items are present in the figure legend, table legend, main text, or Methods section.

| n/a | Confirmed | |
|---|---|---|
| ☐ | ☒ | The exact sample size (*n*) for each experimental group/condition, given as a discrete number and unit of measurement |
| ☐ | ☒ | A statement on whether measurements were taken from distinct samples or whether the same sample was measured repeatedly |
| ☐ | ☒ | The statistical test(s) used AND whether they are one- or two-sided<br>*Only common tests should be described solely by name; describe more complex techniques in the Methods section.* |
| ☐ | ☒ | A description of all covariates tested |
| ☒ | ☐ | A description of any assumptions or corrections, such as tests of normality and adjustment for multiple comparisons |
| ☐ | ☒ | A full description of the statistical parameters including central tendency (e.g. means) or other basic estimates (e.g. regression coefficient) AND variation (e.g. standard deviation) or associated estimates of uncertainty (e.g. confidence intervals) |
| ☐ | ☒ | For null hypothesis testing, the test statistic (e.g. *F*, *t*, *r*) with confidence intervals, effect sizes, degrees of freedom and *P* value noted<br>*Give P values as exact values whenever suitable.* |
| ☒ | ☐ | For Bayesian analysis, information on the choice of priors and Markov chain Monte Carlo settings |
| ☒ | ☐ | For hierarchical and complex designs, identification of the appropriate level for tests and full reporting of outcomes |
| ☒ | ☐ | Estimates of effect sizes (e.g. Cohen's *d*, Pearson's *r*), indicating how they were calculated |

*Our web collection on statistics for biologists contains articles on many of the points above.*

## Software and code

Policy information about availability of computer code

| | |
|---|---|
| Data collection | syngo fast View v.1.0 (Siemens Healthcare); ZEN Blue Software (Zeiss microscopy), LAS microscope software (Leica); HyVolution-II software (Scientific Volume Imaging) |
| Data analysis | syngo fastView v.1.0 (Siemens Healthcare); ZEN 2.3 software (Zeiss Microscopy); Imaris imaging analysis software (Oxford Instruments); Photoshop V 21.0.3. (Adobe); QuPath V 0-2-0-m9 (Bankhead et al., 2017); GraphPad Prism 8 (GraphPad Software, Inc.) |

For manuscripts utilizing custom algorithms or software that are central to the research but not yet described in published literature, software must be made available to editors/reviewers. We strongly encourage code deposition in a community repository (e.g. GitHub). See the Nature Research guidelines for submitting code & software for further information.

## Data

Policy information about availability of data

All manuscripts must include a data availability statement. This statement should provide the following information, where applicable:

- Accession codes, unique identifiers, or web links for publicly available datasets
- A list of figures that have associated raw data
- A description of any restrictions on data availability

Primary data can be made available upon request.

# Field-specific reporting

Please select the one below that is the best fit for your research. If you are not sure, read the appropriate sections before making your selection.

☒ Life sciences ☐ Behavioural & social sciences ☐ Ecological, evolutionary & environmental sciences

# Life sciences study design

All studies must disclose on these points even when the disclosure is negative.

| | |
|---|---|
| Sample size | The Resource equation (E=N-B-T) (Mead, 1988) was used for estimation of the initial animal cohort size, with 10 < E < 20, T=3 (genotypes) and B=0. Based on this, N between 13 and 23 animals (per genotype) would be required. We have exceeded this estimate to ensure the robustness and reliability of the phenotype discovered. The analysis was performed on age-matched cohorts. Genotype, age and number of mice are listed in Supplementary Tables 5 and 6. For sample size of in vitro experiments, please see Supplementary Tables 7 and 8. |
| Data exclusions | No data was excluded from the analyses. |
| Replication | To ensure reproducibility of the analyses the following measures were taken:<br><br>1) In vivo independent replicate experiments: a minimum of 4 mutant animals per genotype (from at least two separate litters) were compared to their litter-mate controls, in immunofluorescence analysis experiments. Furthermore, the in vivo ciliary phenotypes were confirmed by 4 different cilia markers (Acetylated tubulin, ARL13B, CLUAP1, gamma tubulin) as well as anti-TRIP6. For hydrocephalus incidence, a total of 689 animals were analysed, listed in Supplementary Table 5.<br><br>2) In vitro independent replicate experiments: the analysis included three independent experiments, each experiment empoyed at least two ciliary markers or more (Acetylated tubulin, ARL13B, CLUAP1, pericentrin, gamma tubulin, IFT88).<br>All attempts at replication were successful. |
| Randomization | Mice were allocated into experimental groups on the basis of genotyping. Cultured cells were randomly allocated into control and Trip6-inhibiting/down-regulating treatments. |
| Blinding | The investigators were not blinded to group allocations. However, the observations were confirmed independently by multiple investigators. Blinding is crucial for identifying subtle differences, but, the hydrocephaus and ciliary defects are strong (the later also a fully penetrant phenotype) thus blinding was not deemed necessary. |

# Reporting for specific materials, systems and methods

We require information from authors about some types of materials, experimental systems and methods used in many studies. Here, indicate whether each material, system or method listed is relevant to your study. If you are not sure if a list item applies to your research, read the appropriate section before selecting a response.

## Materials & experimental systems

| n/a | Involved in the study | |
|---|---|---|
| ☐ | ☒ | Antibodies |
| ☐ | ☒ | Eukaryotic cell lines |
| ☒ | ☐ | Palaeontology |
| ☐ | ☒ | Animals and other organisms |
| ☒ | ☐ | Human research participants |
| ☒ | ☐ | Clinical data |

## Methods

| n/a | Involved in the study | |
|---|---|---|
| ☒ | ☐ | ChIP-seq |
| ☒ | ☐ | Flow cytometry |
| ☐ | ☒ | MRI-based neuroimaging |

## Antibodies

| | |
|---|---|
| Antibodies used | All antibodies and the dilutions at which they have been used are listed in Supplementary Tables 3 (primary antibodies) and 4 (secondary antibodies).<br><br>Primary Antibodies:<br><br>Anti-Acetylated Tubulin, Sigma (T6793), clone 6-11-B-1, lot# 034M4828; Anti-ARL13B, Proteintech (17/11-1-AP), lot# 00040297; Anti-ARL13B, BiCell Scientific Inc (90413); Anti-ß-Catenin, Sigma (C2206), lot# 097K4842; Anti-ß-Catenin, Santa Cruz Biotechnology (sc-59737), clone 12F7, lot# L0211; Anti-CLUAP1, Proteintech (17470-1-AP), lot# 00008770; Anti-gamma tubulin, Sigma (T6557), clone GTU88, lot 065K4761; Anti-N-cadherin, Santa Cruz Biotechnology (sc-7939); Anti-IFT-88, Proteintech (13967-I-AP); Anti-Paxillin, Transd. Lab (P13520), lot# 51564; Anti-Pericentrin, Biolegend (923701), lot# B215567; Anti-S100ß, DAKO (GA504); Anti-Trip6, Santa Cruz Biotechnologies (sc-34976), clone A15, lot# E0807; Anti-ZO1, Abcam (ab59720).<br><br>Secondary antibodies:<br><br>Donkey anti-Mouse IgG (H+L) Highly Cross-Adsorbed Secondary Antibody, Alexa Fluor 546, Life technologies (A10036), lot# 1736962; Donkey anti-Goat IgG (H+L) Cross-Adsorbed Secondary Antibody, Alexa Fluor 546, Life technologies (A11056), lot# 1714714; Donkey anti-Rabbit IgG (H+L) Highly Cross-Adsorbed Secondary Antibody, Alexa Fluor 546, Life technologies (A10040), lot#1640319; Donkey anti-Mouse IgG (H+L) Highly Cross-Adsorbed Secondary Antibody, Alexa Fluor 488, Life technologies (A21202), lot# 1741782; Donkey anti-Goat IgG (H+L) Cross-Adsorbed Secondary Antibody, Alexa Fluor 488, Life technologies (A11055), lot# 1737907; Donkey anti-Rabbit IgG (H+L) Highly Cross-Adsorbed Secondary Antibody, Alexa Fluor 488, Life technologies (A21206), lot# 1723019; Goat anti-Rat IgG (H+L) Highly Cross-Adsorbed Secondary Antibody, Alexa Fluor 488 Invitrogen (A-11006); Rabbit anti-Goat IgG (H+L) HRP-Conjugated Secondary Antibody, Invitrogen (31402);Donkey anti-Rabbit IgG (H+L) Highly Cross-Adsorbed Secondary Antibody, Alexa Fluor 647, Life technologies (A31573), lot# 1693297; Donkey anti-Mouse IgG (H+L) Highly Cross-Adsorbed Secondary Antibody, Alexa Fluor 647, Life technologies (A31571), lot# 1692912. |

| | |
|---|---|
| Validation | Antibodies that had been validated for species (mouse) and relevant applications (immunofluorescence microscopy, western blotting), as per manufacturers' statements, were used in this study. Additional validation of all antibodies used is available on the manufacturers' websites. Titration of all antibodies to determine optimal dilution, fixation conditions, antigen retrieval conditions has been performed in our laboratory. Furthermore, appropriate positive and negative controls (e.g. knock-out, knock-down, appropriate subcellular localization) were included in every experiment. |

## Eukaryotic cell lines

Policy information about cell lines

| | |
|---|---|
| Cell line source(s) | Immortalized murine choroid plexus cell line Z310 (RRID:CVCL_F753) was kindly provided by Professor Zheng, Purdue University. |
| Authentication | The cell line has been characterized by genotyping, morphology, growth assays and expression of the choroidal epithelium-specific marker TTR (Zheng & Zhao 2002). |
| Mycoplasma contamination | Negative |
| Commonly misidentified lines (See ICLAC register) | No commonly misidentified cell lines were used in the study. |

## Animals and other organisms

Policy information about studies involving animals; ARRIVE guidelines recommended for reporting animal research

| | |
|---|---|
| Laboratory animals | Mouse experiments (including embryo isolation), colony maintenance and breeding were conducted in accordance with Directive 2010/63/EU and the regulations of the Thüringen Landesamt für Verbraucherschutz (Thüringen, Germany) with protocols approved by the respective ethics committee (Tierschutzkomission, Thuringian Animal Welfare Committee) and under the oversight of the FLI Animal Welfare Committee. Animals were provided with standard laboratory chow and tap water ad libitum, and kept at constant temperature (21°C), with relative air humidity of 55%±15 and constant light cycle (12hr-light, 12hr-dark). |
| Wild animals | No wild animals were used in the study. |
| Field-collected samples | No field-collected samples were used in the study. |
| Ethics oversight | |

Note that full information on the approval of the study protocol must also be provided in the manuscript.

## Magnetic resonance imaging

### Experimental design

| | |
|---|---|
| Design type | Resting state |
| Design specifications | Scans were performed on a clinical 3T scanner (Magnetom Trio, Siemens Healthcare) Isotropic images were acquired using a Simens SPACE sequence with a constant flip angle. |
| Behavioral performance measures | n/a (euthanised mice were imaged). |

### Acquisition

| | |
|---|---|
| Imaging type(s) | Structural |
| Field strength | 3 Tesla |
| Sequence & imaging parameters | A Siemens SPACE sequence with a constant flip angle was employed to acquire images with a resolution of 0.2 mm × 0.2 mm × 0.16 mm using the following parameters: echo time TE = 125 msec, repetition time TR = 1900 msec, bandwidth = 130 Hz/px, Turbo Factor TF= 65 and integrated fat saturation. |
| Area of acquisition | Whole head scan. |

Diffusion MRI D Used 0 Not used

## Preprocessing

| | |
|---|---|
| Preprocessing software | MRI data were processed using the software syngo fastView. For depiction of multicolored axial images multiplanar reconstructions from the original 3D MRI data set were prepared. |
| Normalization | n/a (the experiment did not include fMRI measurements) |
| Normalization template | n/a (the experiment did not include fMRI measurements) |
| Noise and artifact removal | n/a (the experiment was performed on euthanized, immobilized mice) |
| Volume censoring | n/a (the experiment was performed on euthanized, immobilized mice) |

## Statistical modeling & inference

| | |
|---|---|
| Model type and settings | n/a (the experiment was performed on euthanized mice) |
| Effect(s) tested | n/a (the experiment did not include fMRI measurements or quantifications) |

Specify type of analysis: 0 Whole brain D ROI-based 0 Both

| | |
|---|---|
| Statistic type for inference (See Eklund et al. 2016) | n/a (the experiment did not include fMRI measurements or quantifications) |
| Correction | n/a (the experiment did not include fMRI measurements) |

## Models & analysis

| n/a | Involved in the study |
|---|---|
| 0 | D Functional and/or effective connectivity |
| 0 | D Graph analysis |
| 0 | D Multivariate modeling or predictive analysis |
